# Telomere dysfunction impairs epidermal stem cell specification and differentiation by disrupting BMP/pSmad/P63 signaling

**DOI:** 10.1371/journal.pgen.1008368

**Published:** 2019-09-13

**Authors:** Na Liu, Yu Yin, Haiying Wang, Zhongcheng Zhou, Xiaoyan Sheng, Haifeng Fu, Renpeng Guo, Hua Wang, Jiao Yang, Peng Gong, Wen Ning, Zhenyu Ju, Yifei Liu, Lin Liu

**Affiliations:** 1 State Key Laboratory of Medicinal Chemical Biology, Nankai University, Tianjin, China; 2 Key Laboratory of Bioactive Materials, Ministry of Education, Department of Cell Biology and Genetics, College of Life Sciences, Nankai University, Tianjin, China; 3 School of Medicine, Nankai University, Tianjin, China; 4 Yunnan Key Laboratory of Primate Biomedical Research; Institute of Primate Translational Medicine, Kunming University of Science and Technology, Kunming, China; 5 Key Laboratory of Regenerative Medicine of Ministry of Education, Guangzhou Regenerative Medicine and Health Guangdong Laboratory, Institute of Aging and Regenerative Medicine, Jinan University, Guangzhou, China; 6 Yale Fertility Center and Department of OB/GYN, Yale University School of Medicine, New Haven, CT, United States of America; Stanford University, UNITED STATES

## Abstract

Telomere shortening is associated with aging and age-associated diseases. Additionally, telomere dysfunction resulting from telomerase gene mutation can lead to premature aging, such as apparent skin atrophy and hair loss. However, the molecular signaling linking telomere dysfunction to skin atrophy remains elusive. Here we show that dysfunctional telomere disrupts BMP/pSmad/P63 signaling, impairing epidermal stem cell specification and differentiation of skin and hair follicles. We find that telomere shortening mediated by *Terc* loss up-regulates *Follistatin* (*Fst*), inhibiting pSmad signaling and down-regulating *P63* and epidermal keratins in an ESC differentiation model as well as in adult development of telomere-shortened mice. Mechanistically, short telomeres disrupt PRC2/H3K27me3-mediated repression of *Fst*. Our findings reveal that skin atrophy due to telomere dysfunction is caused by a previously unappreciated link with Fst and BMP signaling that could be explored in the development of therapies.

## Author summary

Patients with mutations in the telomerase component (eg, Dyskerin, TERT, TERC) are frequently accompanied by symptoms of abnormal epidermis, such as hyperpigmentation, premature skin degradation, hair follicle shedding, skin atrophy, and dry skin. Mice with mutations in telomere-associated proteins or telomerase genes also show similar phenotypes, associated with telomere shortening. However, the underlying molecular signaling and mechanisms remain elusive. Here, we show that the differentiation of epidermis is disrupted resulting from short telomeres. Epidermal differentiation abnormalities can be rescued as the telomere length is extended. Furthermore, we uncover that Fst-BMP-Smad pathway is implicated in regulation of epidermal differentiation by telomeres length.

## Introduction

Telomeres consist of (TTAGGG)n DNA repeats and associated proteins that locate at chromosome ends, maintaining chromosomal stability and cell proliferation. The telomerase complex consists of a telomerase RNA component (TERC) and the reverse transcriptase catalytic subunit (TERT), and adds telomere repeats to chromosome ends to offset the loss of telomere sequences that occurs due to the end-replication problem, the inability of DNA polymerase to replicate fully the lagging DNA strand [1]. In the absence of telomerase, telomeres shorten progressively with cell division, ultimately leading to loss of telomere protection and a DNA damage response that induces senescence or cell death. Telomere shortening is closely tied to organism aging and premature aging and associated diseases [2–5]. Skin atrophy and hair loss are general phenomena associated with age [6]. Moreover, patients with the mutation of telomerase components (e.g. *Dyskerin*, *TERT*, *TERC*) exhibit telomere shortening and skin atrophy [7]. It has been shown that short telomeres impair differentiation and development of the epidermis, and cause skin atrophy and loss of hair follicles, in association with epidermal stem cell dysfunction with aging [8–10]. However, the molecular signaling underlying short telomeres-associated skin atrophy or degeneration and hair follicle loss remains elusive.

Embryonic stem (ES) cells are able to spontaneously differentiate into three embryonic germ layers ectoderm, mesoderm, and endoderm by standard test of embryoid body (EB) formation. This method has been extensively used to investigate signaling pathways that control ES cell differentiation towards various cell lineages [11–13], including epidermis [14–16]. Telomere length is critical for developmental pluripotency and differentiation capacity of ES cells or iPS cells [17–20]. We attempted to investigate how short telomere compromises epidermal lineage specification and differentiation initially by using ES cell lines with different telomere lengths, derived from *Terc* knockout (*Terc*^*–/–*^) mice [17]. We showed that telomere lengths affected differentiation of ES cells into epidermis. We further validated that short telomeres impeded epidermal differentiation in the adult telomerase-deficient, telomere shortened mice. Moreover, we investigated potential regulatory mechanisms of telomere length on epidermis differentiation.

## Results

### Short telomere impairs epidermal stem cell specification and differentiation *in vitro*

**Fig 1:**
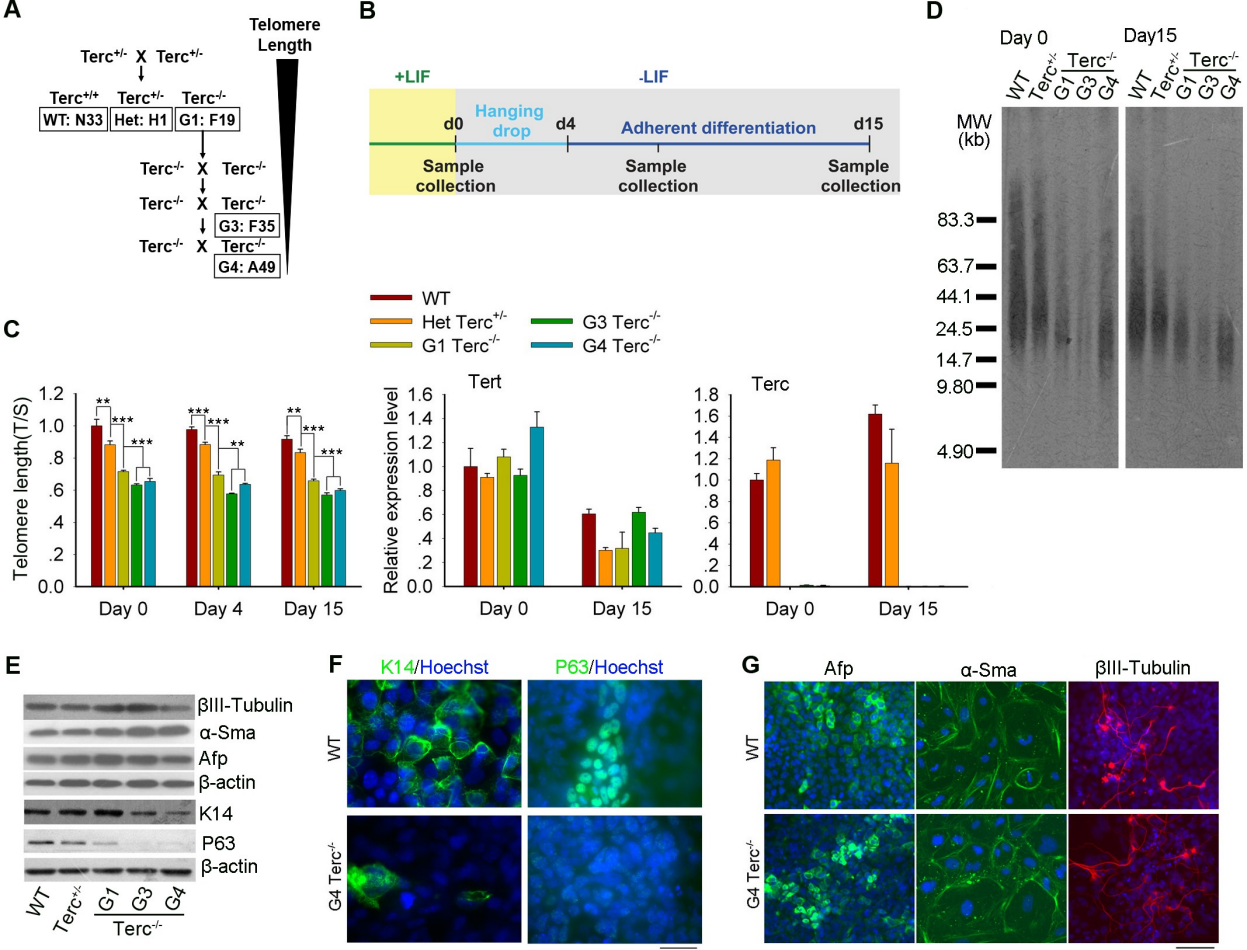
Short telomeres impair differentiation of ES cells into epidermis lineage *in vitro*. (A) Breeding strategy for generating G1, G3, G4 *Terc*^–/–^mice and isolation of their ES cell lines from the corresponding mice. ES cell lines used include WT ES cells (N33), *Terc*^+/-^ ES cells (H1), G1 (F19), G3 (F35), and G4 (A49) *Terc*^–/–^ES cells with long to shortest telomeres, respectively. (B) Schematic illustration of *in vitro* differentiation protocol of ES cells. ES cells were cultured in medium without LIF as hanging drop for 4 days, and then transferred to microwell plates for 11 days. Samples were collected at day 0, day 8, and day 15 following differentiation for various analysis. (C) Telomere length shown as T/S ratio and relative expression levels of *Tert* and *Terc* analyzed by real-time qPCR at day 0, day 4, and day 15 of differentiation. Bars = Mean ± SEM (n = 4). **, p<0.01, ***, p<0.001, compared to WT ES cells at the same time point. (D) Telomere length distribution shown as TRF by Southern blot analysis of ES cells at day 0 and day 15 of differentiation. (E) Protein levels of epidermal (K14 and P63), neural ectodermal (βIII-Tubulin), mesodermal (α-Sma), and endodermal (Afp) markers in ES cells with different telomeres length verified by Western blot analysis at day 15 of differentiation. β-actin served as loading control. (F) Immunofluorescence of epidermal markers K14 and P63 at day 15 of differentiation, displaying areas with defective expression of K14 and P63 in G4 *Terc*^–/–^cells, compared with WT cells. Scale bar = 20 μm. (G) Immunofluorescence of neural ectodermal (βIII-Tubulin), mesodermal (α-Sma), and endodermal (Afp) markers at day 15 of differentiation in G4 *Terc*^–/–^cells and WT cells. Scale bar = 50 μm. ES cells, embryonic stem cells; WT, wild type; K14, Keratin 14.

To investigate the differentiation defects associated with short telomeres, we initially performed *in vitro* differentiation experiments by standard EB formation test using mouse ES cells with various telomere lengths due to telomerase (*Terc*^*–/–*^) deficiency (Fig 1A and 1B, S1A Fig). Telomeres were longest in wild-type (WT) ES cells, shorter in heterozygous (*Terc*^+/–^) and early generation (G1) *Terc*^–/–^ES cells, and critically short or lost in late generation (G3 and G4) *Terc*^–/–^ES cells (Fig 1C and 1D), as we previously reported [17]. Late generation (G3 and G4) *Terc*^–/–^cells also exhibited short telomeres by day15 of differentiation (Fig 1C and 1D). Upon differentiation, WT ES cells showed significantly reduced expression of pluripotency marker genes such as *Oct4* and *Nanog* (S1B and S1C Fig). However, G3 and G4 *Terc*^–/–^ES cells maintained expression of *Nanog* and *Oct4* at relatively high levels, and low methylation at *Nanog* promoter (S1D Fig), consistent with the finding using *Tert*^–/–^ES cells also with critically short telomeres [18]. Expression levels of genes related to endoderm, mesoderm and neuro-ectoderm did not differ between WT ES cells and ES cells with short telomeres, suggesting that shortening of telomeres does not significantly affect the differentiation of these germ layers (Fig 1E and 1G).

Notably, expression levels of genes important for epidermal ectoderm differentiation were consistently reduced in telomere shortened (G3/G4 *Terc*^*–/–*^) ES cells following differentiation as compared to WT ES cells (S1E and S1F Fig). During mouse embryo development, epidermal progenitors are specified at around embryonic day 8–12 (E8-12), later than that of neural induction. Expression of *Keratin 14* (*K14*) was at very low level on day 8 of differentiation (Day 8) and was sharply increased on day 15 in WT cells. *K14* was also low on day 8 in G3/G4 *Terc*^–/–^cells, but dramatically reduced on day 15, as compared to WT cells. Consistently, expression levels of *K5* (epidermal basal cell marker), *K1* (epidermis marker of skin) and *K4* (epidermis marker in stratified epithelia) [21], in the differentiated G3/G4 *Terc*^–/–^cells were also significantly lower than that in WT cells (S1F Fig).

*p63* as one of the earliest genes for epidermal lineage is expressed as early as E7.5, identifies epidermal keratinocyte stem cells, and is required for epidermal differentiation [22–24]. *p63* also is expressed earlier than does *K14* during differentiation of human ES cells into keratinocytes [15]. Consistently, *p63* expression was detectable in WT, *Terc*^+/–^, and G1 *Terc*^–/–^cells by day 7–8 of differentiation, earlier than that of Keratins, but only minimal in G3/G4 *Terc*^–/–^cells. *p63* level was further increased by day 15 in WT, *Terc*^+/–^, and G1 *Terc*^–/–^cells, but much lower in G3 and G4 *Terc*^–/–^cells (S1E Fig). Consistent with the qPCR data, protein levels of both K14 and P63 at day 15 were also greatly reduced in G3/ G4 *Terc*^–/–^cells as compared to WT cells (Fig 1E). Immunofluorescence microscopy showed specific staining of P63 in the nuclei and K14 in cytoplasm and membrane in WT cells but much reduced staining in some G4 *Terc*^–/–^cells (Fig 1F). These data indicated that short telomeres lead to decreased expression of *P63* and *K14* and that telomere-shortened stem cells may fail to stratify in the differentiation into epidermal lineage.

### Short telomere impairs epidermis *in vivo*

**Fig 2:**
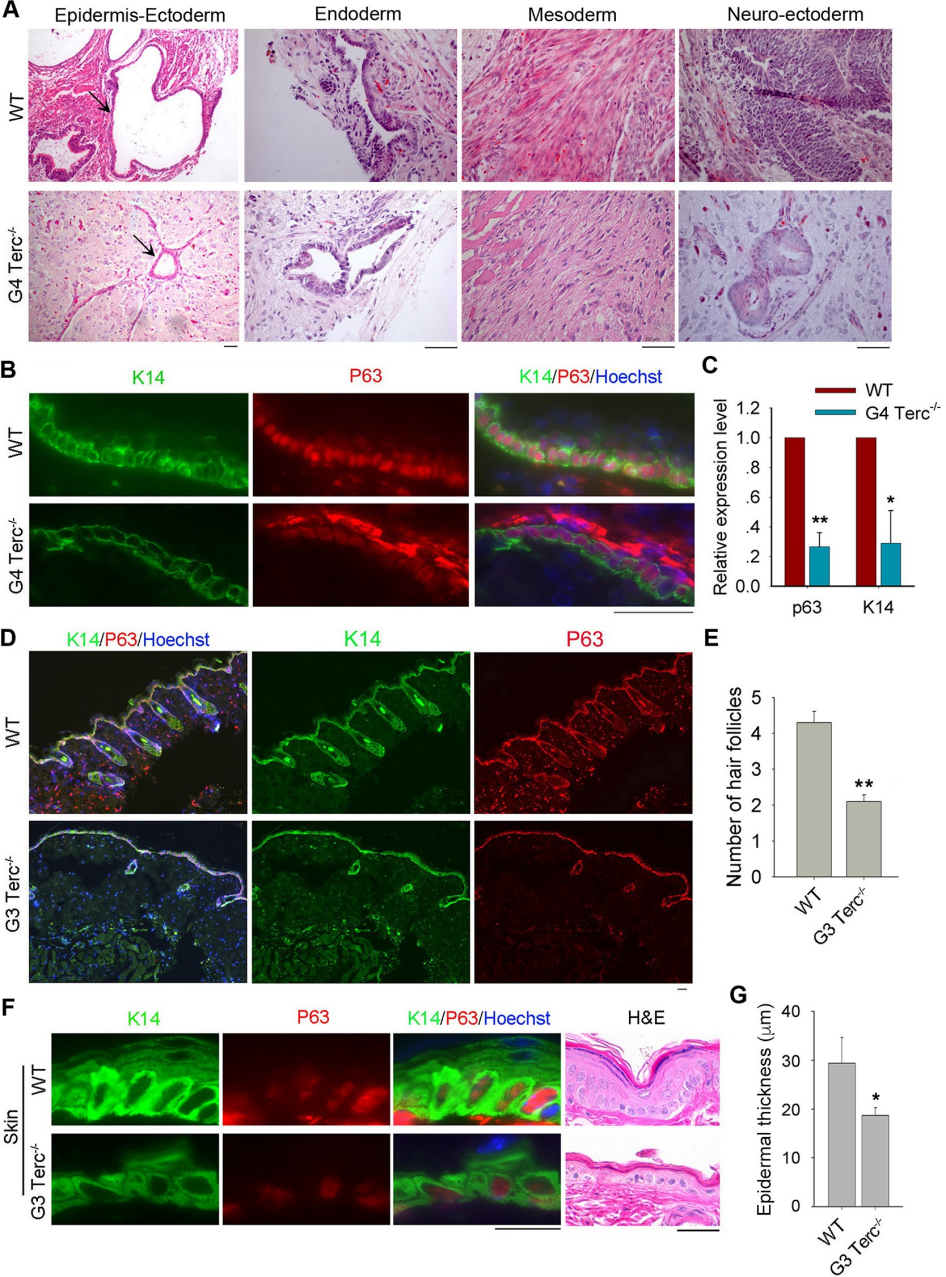
Short telomeres impair epidermal differentiation *in vivo*. (A) Three embryonic germ layers shown by histology following H&E staining of teratomas formed from WT and G4 *Terc*^–/–^ES cells. Scale bar = 50 μm. (B) Immunofluorescence of epidermis markers shown by K14 and nuclear P63 in teratomas formed from WT and G4 *Terc*^–/–^ES cells. Nuclei are stained in blue with Hoechst. Scale bar = 50 μm. (C) Expression levels by qPCR of basal layer markers *K14* and *p63* in teratomas formed from WT and G4 *Terc*^–/–^ES cells. Bars = Mean ± SEM (n = 3). *, p<0.05; **, p<0.01, compared with WT teratomas. (D) Representative immunofluorescence images showing co-staining of P63 with K14 in the sections of mouse skin epidermis. WT mouse skin displays many hair follicles underneath and G3 *Terc*^–/–^mouse skin shows fewer and smaller hair follicles. Scale bar = 25 μm. (E) Number of hair follicles in WT and G3 *Terc*^–/–^mouse skin per field view. Ten field view was counted, **, p<0.01. (F) Representative images showing skin (back) of WT and G3 *Terc*^*–/–*^mice revealed by immunofluorescence of K14 and P63 and histology by H&E staining. Scale bar = 20 μm. (G) Thickness of skin epidermis in WT and G3 *Terc*^–/–^mice estimated from H&E histology. *, p<0.05.

To examine the impacts of short telomeres on the differentiation capacity *in vivo*, we initially performed standard teratoma formation test [12,25]. Both WT and G4 *Terc*^*–/–*^ES cells were able to differentiate into three germ layers, including endoderm, mesoderm, and neural ectoderm revealed by histology (Fig 2A). However, epidermis lineage was reduced in teratomas differentiated from ES cells with short telomeres, in contrast to that of WT ES cells (Fig 2A and 2B, S2A Fig). Structures in size or number identified by epidermis marker K14 or epidermal stem cell marker P63 were reduced in the sections of teratomas from G4 *Terc*^-/-^ ES cells, compared with those from WT ES cells (Fig 2B). Relative mRNA levels of *p63* and *K14* in teratomas derived from G4 *Terc*^*–/–*^ES cells also were lower than those from WT ES cells (Fig 2C).

Similar phenotypes also can be observed in the adult G3 *Terc*^*–/–*^mouse skin. Epidermis marked by co-immunostaining of P63 and K14 and by histology was thinner on average in skin of two-three month old G3 *Terc*^*–/–*^mice, compared with age-matched WT mice (Fig 2F and 2G, S2B and S2C Fig). Additionally, hair follicles were readily seen in dermis of WT mice but fewer in G3 *Terc*^*–/–*^mice (only 50% of WT mice) (Fig 2D and 2E, S3B Fig). Number of hair follicles was calculated based on at least 10 fields of view under microscopy. In WT mouse skin, the hair follicles are structurally intact with an average of 4 to 5 per field of view. However, hair follicles drop sharply in their numbers and loses the typical structure in the G3 *Terc*^*–/–*^mouse skin (Fig 2D and 2E). Both *in vitro* and *in vivo* results validated that short telomeres reduce epidermal commitment.

### Short telomere leads to excessive expression of *Fst* and represses BMP/pSmad signaling

**Fig 3:**
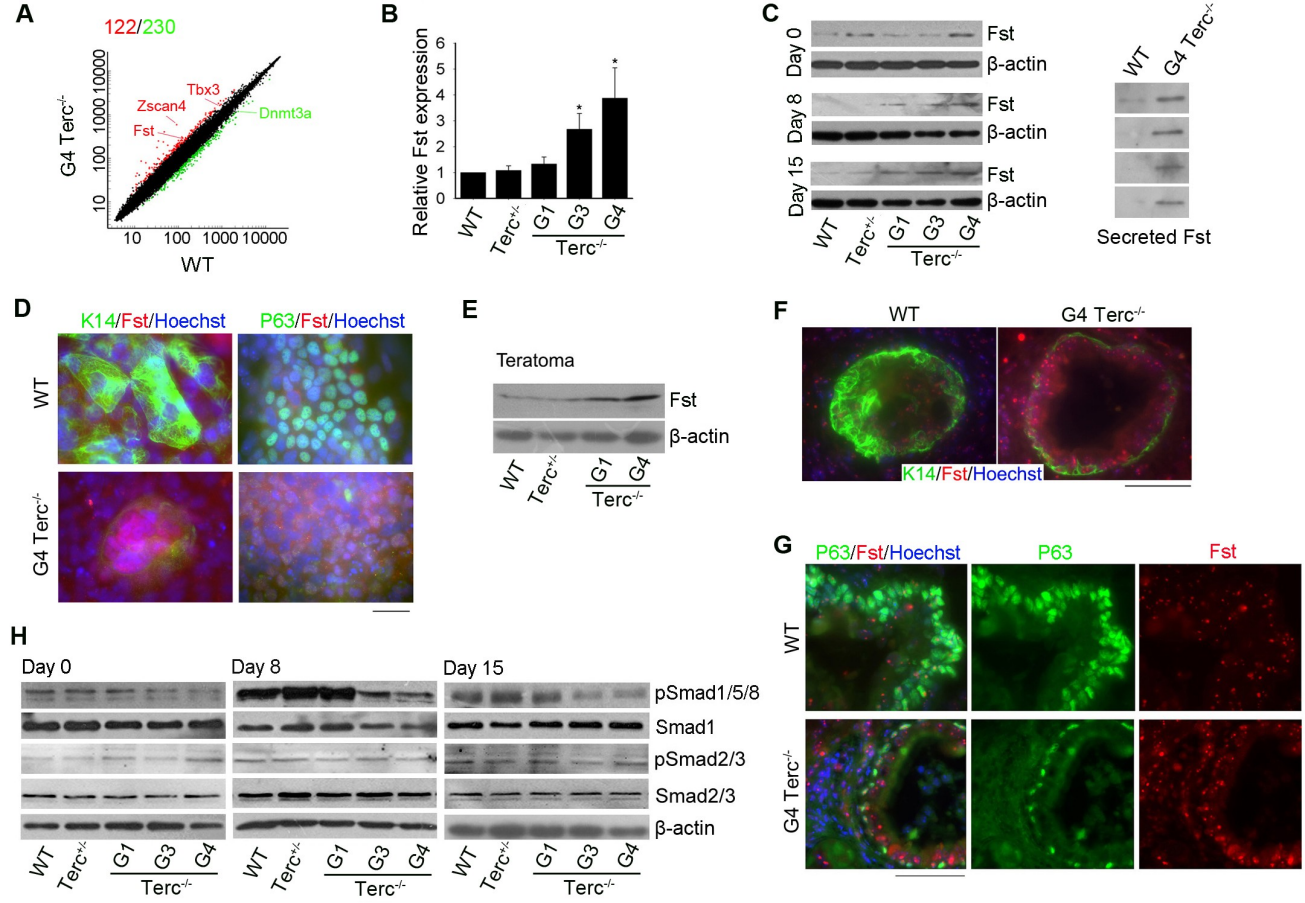
Telomere length regulates Fst/BMP/pSmad signaling. (A) Scatter plots showing global differential gene expression profile of WT and G4 *Terc*^–/–^ES cells. At least 1.8-fold change was used as cut-off for differentially expressed genes. Red, up-regulated genes and green, down-regulated genes in G4 *Terc*^–/–^cells relative to WT cells. Both axes (in log^10^ scale) represent the normalized gene expression values averaged from duplicates. (B) *Fst* expression level in ES cell lines determined by qPCR, normalized to *Gapdh* and expressed as relative expression to WT ES cells. Bars = Mean ± SEM (n = 3). *, p<0.05. (C) Protein levels of Fst at day 0, day 8 and day 15 of differentiation of ES cells analyzed by western blot. β-actin levels in cells served as loading control. (D) Expression of Fst (red) and co-staining with K14 (green) or P63 (green) in ES cells and differentiated cells revealed by immunofluorescence microscopy. Fst distributed inside and around the cells was expressed at higher levels in G4 *Terc*^–/–^cells than in WT cells, and inversely correlated with decreased expression of nuclear P63 and cytoplasmic or membrane K14 in ES cells and following differentiation. Nuclei are stained in blue with Hoechst 33342. Scale bar = 20 μm. (E) Western blot of Fst protein level in the teratomas differentiated from WT, *Terc*^+/–^, G1, and G4 *Terc*^–/–^ES cells. (F) Immunofluorescence of K14 (green) and Fst (red) in teratomas from WT and G4 *Terc*^–/–^ES cells. Scale bar = 50 μm. (G) Representative immunofluorescence images showing co-staining of P63 (green) with Fst (red) in sections of teratoma. Scale bar = 50 μm. (H) Analysis of protein levels of Smad and pSmad by western blot during differentiation. β-actin levels in cells served as loading control. Fst, follistatin; BMP, bone morphogenetic protein.

To understand the mechanisms underlying short telomeres-affecting ES cell differentiation towards epidermal lineage, we performed microarray analysis of G4 *Terc*^*–/–*^ES cells compared with WT ES cells. Interestingly, *Follistatin* (*Fst*), a negative regulator of Smad pathway which is critical in epidermis commitment, was expressed at higher level in G4 *Terc*^*–/–*^than in WT ES cells at day 0 (Fig 3A). qPCR analysis validated that expression levels of *Fst* were higher in G3 and G4 *Terc*^–/–^than in WT ES cells (Fig 3B). Western blot also confirmed that Fst protein level was indeed higher in G4 *Terc*^*–/–*^ES cells than in WT ES cells during differentiation (Fig 3C, left panel). Given that Fst is a secreted protein, we also examined Fst protein levels in the culture media for both cell lines. Fst protein was highly abundant in the culture media of G4 *Terc*^*–/–*^ES cells, but barely detectable in that of WT ES cells (Fig 3C, right panel). Furthermore, robust cytoplasmic and membrane staining of K14 and nuclear P63 were observed at day 15 in differentiated WT cells, but their expression levels were markedly reduced in differentiated G4 *Terc*^*–/–*^cells, where higher Fst fluorescence signals with dotted staining still were readily visible in the cytoplasm of or around the differentiated cells, compared with lower Fst fluorescence in WT cells (Fig 3D). Additionally, Fst protein level was higher in G4 *Terc*^–/–^teratomas than in control teratomas (WT and *Terc*^+/–^) (Fig 3E). Compared to WT teratomas, G4 *Terc*^–/–^teratomas exhibited strong Fst immunofluorescence spotted inside or outside the cells, coincided with less and weak fluorescence staining of K14 and P63 (Fig 3F and 3G). These data provide further evidence that higher expression level of *Fst* is linked to short telomere.

Similar results were obtained from the skin of adult mice. Two-three month old G3 *Terc*^–/–^mice displayed thinner epidermis and skin atrophy compared to the age-matched WT mice, consistent with previous studies [8,26]. K14 level also was reduced in the epidermis of G3 *Terc*^*–/–*^adult mice, accompanied by increased expression of spotty Fst as compared to WT mouse epidermis (S3A Fig). Immunofluorescence staining of Fst in teratomas or tissues appears to be dotted in pattern, somewhat different from the immunostaining in cultured cells, probably because Fst can be locally confined with specific structure in tissues, whereas it diffuses in and around the cultured cells. In addition, G3 *Terc*^–/–^mice displayed defective hair follicle development as evidenced by notably reduced number of hair follicles, with reduced expression of K14 and increased Fst, as well as impaired bulb and bulge at the basal follicles where progenitor cells reside, in contrast to the intact bulb (hair germ) and bulge in WT mice (S3B Fig). Taken together, short telomeres lead to excessive expression of *Fst*, which is incompatible with epidermal stem cell specification and stratification of skin and hair follicles.

### Fst negatively regulates pSmad1/5/8 and *p63*

Collectively, these findings suggested that short telomere specifically prevents the transition from the common ectodermal progenitor state into the epidermis fate. Bone morphogenesis protein 4 (BMP4) signaling is known to be activated in the embryo at the time of ectodermal fate determination, inhibits premature neural differentiation while inducing epidermis development, and can act through phosphorylation and nuclear accumulation of Smad1/5/8 [27–30]. Differentiation of epidermal cells appears to be controlled, in part, by BMP4 [31]. Fst is an antagonist of BMP4. We next asked if the Fst-BMP-Smad1/5/8 signaling pathway plays a critical role in epidermal differentiation. In the wild-type ES cells, the up-regulation of BMP4, BMP7, Smad1, and the down-steam target (Gata1) during differentiation indicates that this pathway plays an important role in normal differentiation and development of epidermis (S4 Fig).

Compared with those of WT, *Terc*^*+/–*^, or G1 *Terc*^*–/–*^ES cells, levels of phosphorylated Smad1/5/8 were reduced in G3/G4 *Terc*^*–/–*^ES cells during differentiation (Fig 3H), suggesting that this pathway is suppressed by short telomeres. Gene expression upstream of this pathway seemed to be not affected by short telomeres. Downstream target genes of this signaling pathway such as Gata1 were expressed at lower levels in G4 *Terc*^*–/–*^than in WT cells by day 8 and day 15 of differentiation (S4 Fig). These data suggested that short telomeres suppress BMP/pSmad signaling following differentiation.

**Fig 4:**
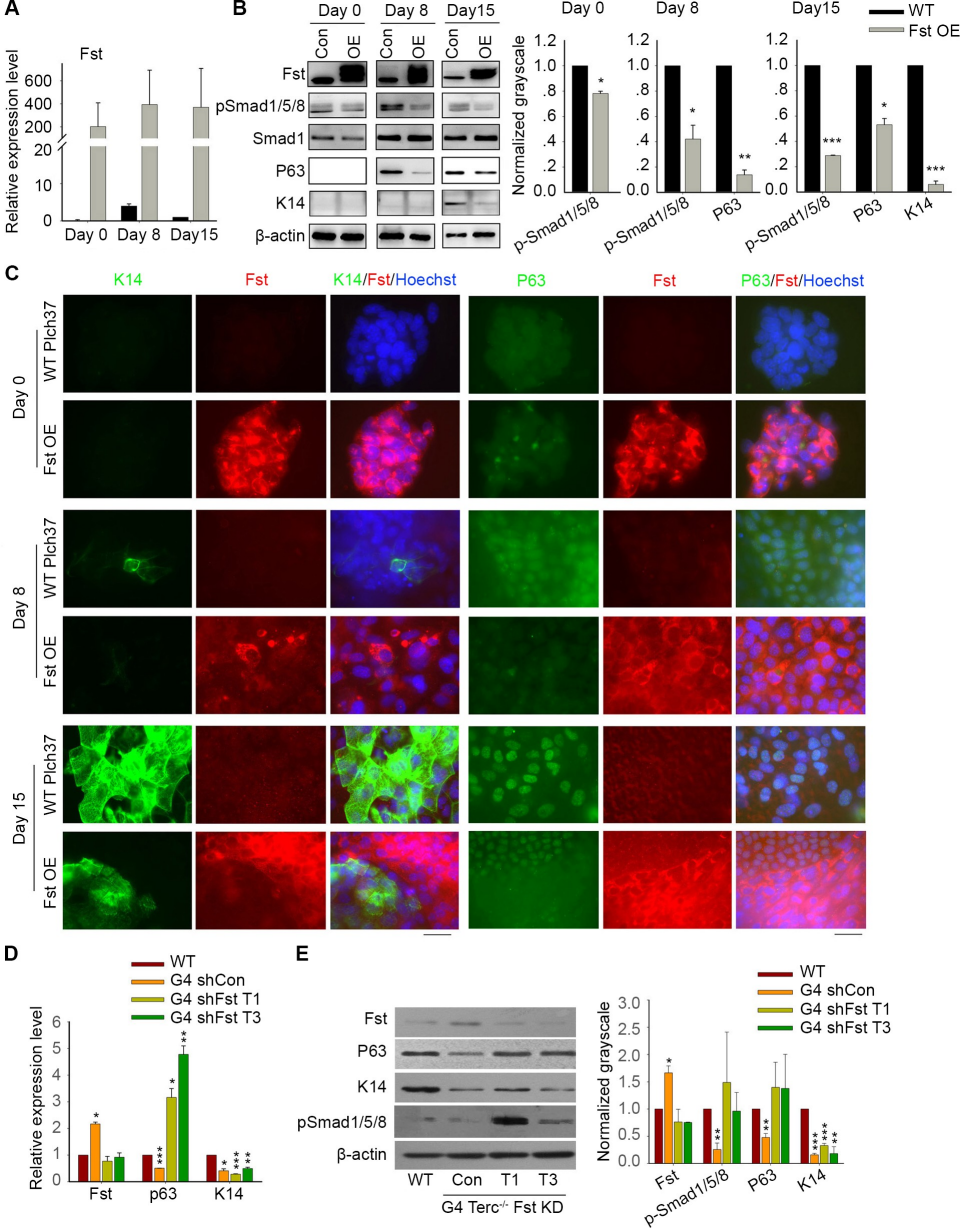
Fst inhibits pSmad, P63 and K14. (A) Relative expression levels by qPCR of *Fst* during differentiation of WT ES cells stably overexpressing *Fst* (OE), compared with WT ES cells transfected with empty vector served as controls (Con). Bars = Mean ± SEM (n = 3). (B) Protein levels of Fst, Smad, pSmad, P63 and K14 by Western blot in *Fst* overexpressed ES cells compared with controls. Right panel, quantification of proteins level using ImageJ software, normalized to β-actin. *, p<0.05; **, p<0.01; ***p<0.001, compared to controls. (C) Overexpression (OE) of *Fst* in ES cells decreases expression of P63 and K14 by immunofluorescence microscopy. While WT ES cells transfected with construct Plch37 served as control express Fst at only a low level, ES cells stably overexpressing *Fst* express Fst at a much higher level by immunofluorescence microscopy. Following differentiation of ES cells overexpressing *Fst*, immunofluorescence staining of cytoplasmic K14 and nuclear P63 is dramatically reduced, compared with that of Plch37 plasmid controls. Scale bar = 20 μm. (D&E) Knockdown (KD) of *Fst* in G4 *Terc*^–/–^cells at day 15 of differentiation leads to increased mRNA levels of *P63* by qPCR (D) and also elevated protein levels of pSmad1/5/8 and P63 by Western blot (E). Right panel, quantification of protein levels using ImageJ software, normalized to β-actin. *, p<0.05; **, p<0.01; ***p<0.001, compared to WT. T1 and T3 are two independent interference RNA sequences targeting *Fst*.

Above data imply that elevated expression of *Fst* resulting from short telomere might lead to reduction of pSmad1/5/8, P63 and K14, and thus defective epidermal stem cell specification and differentiation. To further validate this concept, we generated *Fst* overexpression (OE) ES cell line (Fig 4A) and performed EB differentiation test using WT ES cell line as control. Western blot showed that *Fst* OE ES cells expressed p63 and K14 at reduced levels on day 8 and day 15 of differentiation, which was also confirmed by immunofluorescence microscopy (Fig 4B and 4C). Notably, in the differentiated *Fst* OE cell culture, areas with intensive Fst fluorescence indicative of high expression level exhibited minimal K14 staining, and yet areas with low Fst fluorescence displayed strong K14 or p63 staining (Fig 4C). Hence, high Fst level is discordant with expression of P63 and K14. Consistently, pSmad1/5/8 was decreased in *Fst* OE cells (Fig 4B). These data suggest a conserved but new role of *Fst* in negatively regulating pSmad-signaling pathway during epidermal ectoderm induction.

To test whether reducing *Fst* can de-repress down-stream genes/signaling for epidermis, we knocked down *Fst* by RNA interference in the differentiated G4 *Terc*^*–/–*^cells. Effective knockdown of *Fst* by shRNA in differentiated G4 *Terc*^*–/–*^cells up-regulated the levels of pSmad1/5/8 and P63 (Fig 4D and 4E). Fst downgregulation by RNA interference in the differentiated G4 *Terc*^*–/–*^cells rescued P63 but not fully rescued K14 expression. This may be explained by three potential reasons. Changes in the expression level of K14 could be delayed following P63 expression during epidermal differentiation. Factors other than Fst alone also might be involved in regulation of K14 expression. Alternatively, the regulation of Fst-P63-K14 may slightly differ in differentiated ES cells compared with undifferentiated ES cells as model. Nevertheless, these data further support the notion that excessive expression levels of *Fst* negatively regulate pSmad1/5/8 signaling and *p63*, weakening epidermal stem cell specification and differentiation.

### Repair of *Terc* rejuvenates telomeres and rescues Fst/P63/K14 signaling

**Fig 5:**
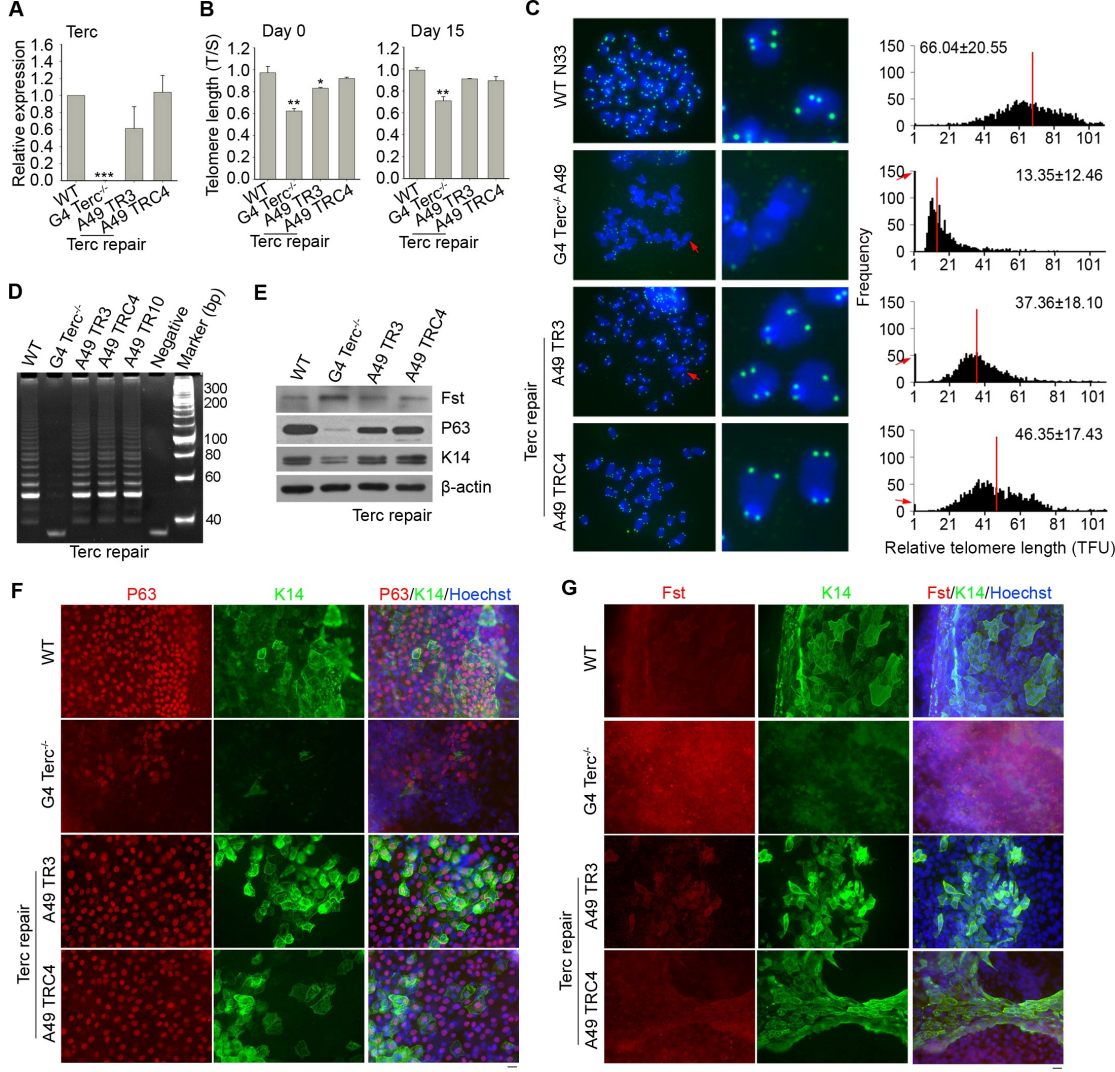
Repair of *Terc* rejuvenates telomeres and partly normalizes Fst/P63 signaling. (A) Expression level of *Terc* by real-time qPCR after *Terc* repair. ***, P<0.001. (B) Relative telomere length shown as T/S ratio by real-time qPCR after *Terc* repair. *, p<0.05; **, p<0.01. (C) Telomere quantitative FISH images and histogram showing relative telomere length distribution as telomere fluorescence intensity unit (TFU). n = 10–15 spreads analyzed for each cell line. Red arrows indicate telomere loss or chromosome fusion. Red line indicates medium telomere length. (D) Telomerase activity measured by TRAP assay. Lysis buffer served as a negative control. (E) Protein levels by Western blot analysis of P63 and K14 in *Terc* repaired cells at day 15 of differentiation. β-actin served as loading control. (F&G) Immunofluorescence of K14 and P63 (F), or Fst and K14 (G) at day 15 of differentiation of WT ES cells, G4 *Terc*^–/–^ES cells, and *Terc*-repaired G4 *Terc*^–/–^ES cells.

Then, we tested whether rejuvenating telomeres in ES cells with short telomeres can repress *Fst*. Using CRISPR/Cas9 technology, we successfully knocked in *Terc* in G4 *Terc*^–/–^ES cells and obtained several *Terc*-repaired ES cell lines (two lines are shown in Fig 5A). These *Terc* repaired (TR) ES cell lines exhibited much longer telomeres than did their parental G4 *Terc*^–/–^ES cell line after culture for 10 passages. Yet, their telomeres were still shorter than those of WT cells as revealed by qPCR and QFISH (Fig 5B and 5C), presumably because of inadequate passages, even though the telomerase activity was recovered (Fig 5D). Frequency of telomere loss was significantly reduced in *Terc* repaired ES cell lines, in contrast to that of G4 *Terc*^–/–^ES cells (Fig 5C). We repeated the *in vitro* differentiation assay with WT, G4 *Terc*^–/–^, and *Terc* repaired ES cell lines. On day 15 of differentiation, telomere length was also rescued in *Terc* repaired cells compared with *Terc*^–/–^cells (Fig 5B). *Terc* repaired cells showed reduced level of Fst and noticeably increased protein levels of P63 and K14 as compared to those of G4 *Terc*^–/–^cells (Fig 5E), which were confirmed by immunofluorescence microscopy (Fig 5F and 5G). These results suggested that epidermal differentiation could be rescued by repairing *Terc* and restoration of telomere length.

### *Fst* is regulated by PRC2-mediated repression

**Fig 6:**
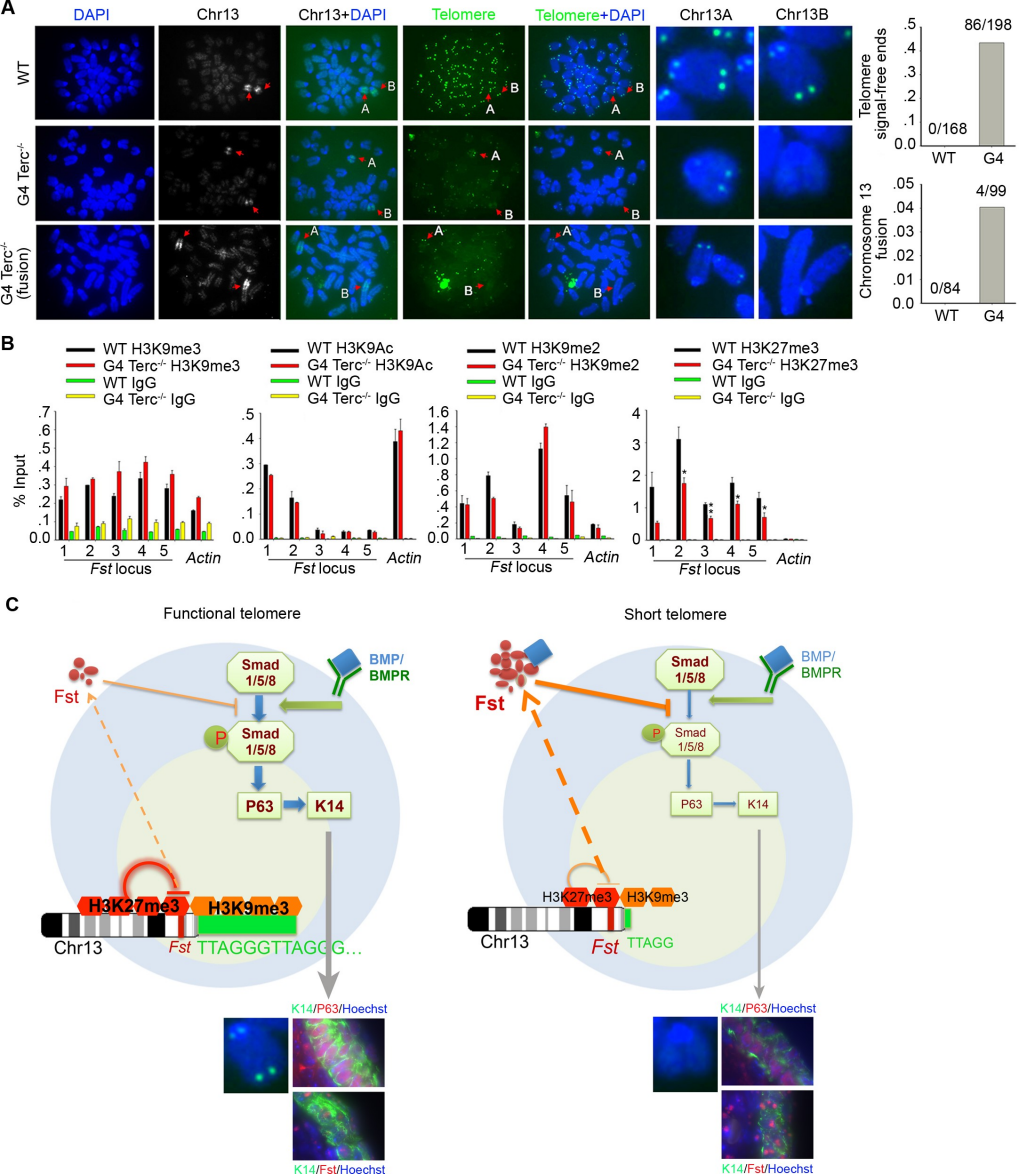
*Fst* is regulated by epigenetic modification. (A) Frequency of telomere signal-free ends and fusion of chromosome 13 in G4 *Terc*^–/–^ES cells, compared with WT ES cells. Telomere FISH by PNA probe and chromosome identification by XMP13 probe of WT and G4 *Terc*^–/–^ES cells. Arrows indicate chromosome 13 stained with XMP13. Chr13A and Chr13B are a pair of chromosome 13 in the same spread. Loss of telomeres near *Fst* gene locus and fusion of chromosome 13 are compared between WT and G4 *Terc*^–/–^ES cells. (B) ChIP-qPCR analysis of abundance of H3K9me3, H3K9me2, H3K9Ac, and H3K27me3 at *Fst* promoter loci in WT and G4 *Terc*^–/–^ES cells. Mean ± SEM (n = 3). *, p<0.05; **, p<0.01, compared to WT cells. (C) A simplified model showing regulation by telomere length of Fst/BMP4-Smad/P63 signaling in epidermal stem cell specification and differentiation. With functional telomeres, enrichment of PRC2/H3K27me3 at *Fst* promoter foci represses *Fst*, maintaining normal BMP4-Smad signaling and proper expression levels of P63 and Keratins (e.g. K14), in the specification and differentiation of epidermis and hair follicles. In the event of telomere shortening or loss, abundance of H3K27me3 at *Fst* foci is reduced, and this causes increased expression of *Fst*, which impairs BMP4-pSmad signaling, and consequently reduces P63 and K14 expression, epidermal stem cell specification and differentiation.

The critical question was how short telomeres result in excessive *Fst* expression. *Fst* gene is located at the subtelomere region of the long arm of chromosome 13, whose expression might be regulated by telomere position effect (TPE) [32]. To reveal the telomere state of chromosome 13, we performed immunofluorescence microscopy to detect the chromosome 13 using the chromosome specific probe followed by telomere FISH. Notably, one pair of chromosome 13 in G4 *Terc*^*–/–*^ES cells constantly displayed telomere signal-free ends, indicative of telomere loss, in contrast to four intact telomere signals of WT ES cells (Fig 6A). Moreover, chromosome fusion or translocation was found in the chromosome 13 with loss of telomere signals in G4 *Terc*^*–/–*^ES cells (Fig 6A).

Loss of telomeric repeats leads to a change in the heterochromatic architecture with decreased H3K9me3 abundance at telomeres/subtelomeres [33]. We tested whether epigenetic modifications are implicated in regulation of *Fst*. Both DNA methyltransferases Dnmt3a and 3b were expressed at lower levels in G4 *Terc*^–/–^ES cells than in WT cells, but Dnmt3b expressed at higher levels following differentiation (S5A Fig). By ChIP-qPCR analysis using specific primers and Dnmt3b antibody, levels of Dnmt3b at *Fst* loci did not differ between G4 *Terc*^–/–^and WT ES cells (S5B Fig). Also, *Fst* promoter showed only low methylation levels in G4 *Terc*^–/–^and WT ES cells like that of MEF (S5C Fig). Methylation levels at subtelomeres of chromosome 13 were greatly reduced in G4 *Terc*^–/–^ES cells, but markedly increased in G4 *Terc*^–/–^cells following differentiation, compared with WT ES cells (S5D Fig). These data suggest that *Fst* promoter methylation may not directly contribute to excessive *Fst* expression due to short telomere.

We analyzed the abundance of histone modifications of H3K4me3, H3K9me3 and H3K27me3 by western blot. H3K4me3 abundance seemed not to differ between G4 *Terc*^–/–^and WT ES cells, while H3K9me3 and H3K27me3 abundance were slightly reduced in G3/G4 *Terc*^*–/–*^ES cells, compared with WT, heterozygous, or G1 *Terc*^–/–^ES cells (S6A Fig). Also, G4 *Terc*^–/–^ES cells exhibited decreased H3K9me3 immunofluorescence and foci at heterochromatin and telomeres prior to and after differentiation, compared with WT ES cells (S6B Fig).

Furthermore, we performed ChIP-qPCR analysis to examine the abundance of H3K9me3/2, H3K9ac and H3K27me3 at *Fst* promoter loci using β-actin as a control. Enrichment of H3K9me3, H3K9Ac, and H3K9me2 at *Fst* promoter was low and showed no significant difference between WT and G4 *Terc*^–/–^ES cells (Fig 6B). However, H3K27me3 was highly enriched at *Fst* promoter. Importantly, H3K27me3 level was markedly reduced at all five loci of Fst promoter in G4 *Terc*^–/–^, compared with that of WT ES cells (Fig 6B). By luciferase reporter assay, the *Fst* promoter activity was higher in G4 *Terc*^–/–^than in WT ES cells (S7A Fig). We further examined expression levels of *Eed*, *Suz12*, *Ezh1* and *Ezh2* which are catalytic components of Polycomb repressive complex PRC2 and potentially tri-methylate H3K27 to repress gene expression and that are shown to play important roles in skin stem cell function and differentiation [34,35]. Expression levels of *Ezh1* and *Ezh2* are reduced in G4 *Terc*^–/–^ES cells as compared to WT ES cells (S7B Fig). Telomere-repaired ES cells partially restored Ezh1/2 expression, together with increased H3K27me3 enrichment at *Fst* promoter (S7C and S7D Fig). Pluripotent marker genes Nanog and Oct4 were also down-regulated during differentiation of *Terc* repaired G4 ES cells, like those of WT ES cells (S7E and S7F Fig). It is interesting to note that *Ezh2* expression level in one *Terc*-repaired ES cell line (A49 TR3) was not recovered well, and coincidently this clone had relatively shorter telomere than that of WT ES cells (Fig 5C). These results further suggest that short telomeres reduce H3K27me3 enrichment at *Fst* promoter, likely together with reduced Ezh1 and Ezh2 levels, de-repress *Fst*, and these together may contribute to excessive expression of *Fst*. Excessive Fst further down-regulates p63/K14 through disrupting BMP4/pSmad signaling (Fig 6C).

## Discussion

Based on the data obtained from both ES cell differentiation *in vitro* and *in vivo*, we propose that functional telomere is important for suppressing *Fst* to prevent its overexpression and to maintain normal expression of P63 and K14 during epidermal stem cell specification and differentiation. Short telomere disrupts PRC2- H3K27me3-mediated repression of *Fst*, which leads to excessive *Fst* expression. Consequently, excessive Fst suppresses BMP/pSmad signaling, reducing P63 and keratins and resulting in epidermal differentiation defects and skin atrophy. This model links dysfunctional telomeres to skin atrophy and hair follicle loss by disrupting Fst/BMP/pSmad/P63/K14 signaling.

This study also provides additional evidence in supporting that ES cell differentiation model is a powerful alternative tool to discover novel signaling and mechanisms that are involved in *in vivo* cell lineage specification at very early developmental stages that might not be readily revealed in live mouse model and particularly in humans [11]. The differentiation assay used in our study shows that the dynamics of P63 and K14 in mouse ES cell is similar to that of human ES cells and mouse embryonic skin development [14]. Our results also confirmed that P63 is a master regulator for K14, K5 and other epidermal genes [36,37], and that BMP4/pSmad signaling pathway can activate P63 [38]. BMP4 negatively regulates neural induction and promotes epidermogenesis during differentiation of mouse ES cells [31], whereas blocking BMP signaling facilitates differentiation of human ES cells into neural lineages [28].

Mounting evidence supports the notion that telomere dysfunction is accompanied by symptoms of abnormal epidermis [10,39–42]. Mice with critically short telomeres exhibit symptoms, including epidermal abnormalities such as poor wound healing, ulcerative skin lesions, early hair loss and early hair graying [2,8,10,43]. We show that short telomeres lead to reduced expression of P63 and declined epidermal stratification and formation, linking to skin atrophy. Study of P63-null mice demonstrates important roles of P63 in orchestrating first epidermal stratification [44,45]. p63-null mice exhibit striking defects in embryonic epidermal morphogenesis [46], and also suffer from diminished stem cell renewal capacity [47]. Moreover, TAp63 serves to maintain adult skin stem cells and prevents premature tissue aging [45,48,49]. Hence, P63 is required to maintain epidermal stem cell renewal while allowing K14 expression and epidermal differentiation [24]. Short telomeres cause stem cell failure [50], and also impair the ability of epidermal stem cells to mobilize out of the hair follicle niche, and thus skin and hair growth [26]. On the other hand, hyper-long telomeres are advantageous for skin regeneration compared with normal length telomeres [51]. Our data provide novel molecular mechanisms of linking short telomeres to reduced pSmad signaling and P63 and thus declined epidermal differentiation.

Moreover, excessive *Fst* expression resulting from short telomere negatively regulates BMP/pSmad/P63 pathways in the epidermal stem cell specification and differentiation. It has been reported that Fst is an antagonist of BMPs by blocking binding of BMP with its receptor [52]. Excessive Fst may compete with BMPs and inhibit BMP-pSmad signaling. Telomere re-elongation successfully achieved by CRISPR/Cas9-mediated knock-in of *Terc* represses *Fst* and recovers expression of P63 and K14. Consistently, telomerase reintroduction into mice with critically short telomeres is sufficient to elongate telomeres in skin keratinocytes and to correct epidermal hair follicle stem cell defects, and rescues skin and hair growth defects [26]. These data also may explain the early findings that *Fst*-knockout mice die within hours of birth but show thicker epidermis [53]. Likewise, deletion of *Fst* results in enhanced keratinocyte proliferation in the tail epidermis of these animals and an earlier onset of keratinocyte hyperproliferation at the wound edge after skin injury, suggesting that *Fst* regulates epidermal homeostasis and also wound repair [54].

In agreement, *Fst*-overexpression transgenic mice are characterized by a thinner dermis and epidermis, reduced density of the dermis and smaller hair follicles, indicative of skin atrophy, and a severe delay in wound healing observed after injury [55]. Moreover, mice that overexpress *Fst* are smaller compared with their control littermates, and their body weight is significantly reduced. This phenotype is similar to that of late generation *Terc*^*–/–*^mice [2,56] (also shown in this study), and these mice also exhibit severely impaired wound healing [2]. Coincidently, *p63*^−/−^ mice have an impaired wound-healing response as well [48]. Together, these data support the idea that abnormal *Fst/p63* signaling is implicated in short telomeres-associated skin atrophy and wound healing. Excitingly, mouse ES cells with hyper-long telomeres generate healthier chimera mice that also have longer telomeres and exhibit delayed aging and high capacity for skin wound healing [51].

Short telomeres can change expression of many genes and signaling pathways particularly with cell differentiation, as shown by the transcription profile data. We identified unique alterations of down-stream genes under regulation by the major TGFβ superfamily during differentiation of ES cells into epidermal lineage. Fst happens to be an evident negative regulator upstream in this pathway, and is up-regulated when telomere is short. We searched for mechanisms underlying short telomere-induced activation of *Fst*, and tested the hypothesis that repressive histone modification or DNA methylation may underlie telomere suppression of *Fst*. By ChIP-qPCR assay with selective related antibodies, we show that PRC2-mediated repression involving Ezh1/2 and H3K27me3 makes a major contribution to suppressing *Fst*. In fact, the regulatory region of *Fst* gene is characteristic of bivalent genes whose promoters are enriched for both activating mark by H3K4me3 and repressing mark by H3K27me3 and Ezh2, primed for differential expression upon differentiation [57–59]. In mice, PRC2 has been found to be enriched in the progenitor cells of developing epidermis, regulates epidermal specification in mouse embryos and maintains hair follicle homeostasis [60,61]. H3K27me3 marks are enriched in a subset of epidermal differentiation gene promoters in undifferentiated cells and disappear on a subset of epidermal gene promoters upon differentiation [62]. Moreover, Ezh1 and Ezh2 repress premature differentiation and H3K27me3 is involved in early lineage specification of embryonic epidermis differentiation [60]. Interestingly, Ezh1/2 null skin progenitors show reduced H3K27me3 abundance and significant up-regulation of Fst [61]. Identification of hair follicle stem cell signature genes showed that *Fst* also is one of genes involving transit-amplifying (TA) progeny repressed by H3K27me3, whereas BMP4 signaling is activated during this process likely induced by epigenetic shift to control by H3K4me3 and H3K79me2 [63]. Consistently, short telomere reduces H3K27me3 enrichment at *Fst* promoter, which leads to elevated *Fst* expression and defective epidermal specification and differentiation. *Terc*-repaired G4 *Terc*^*–/–*^ES cells rejuvenate telomere length to various degrees and partly restore H3K27me3-mediated suppression of *Fst*. *Nanog* and *Oct4* are down-regulated following differentiation of *Terc*-repaired G4 *Terc*^*–/–*^ES cells like WT cells. Coincidentally, *Tert*^-/-^ ES cells also have critically short telomeres and disrupted PRC2 function and low H3K27me3 enrichment at *Nanog* promoter, leading to defective suppression of *Nanog* during differentiation [18]. Fst-BMP4 signal pathway is known as a critical regulator for epidermal differentiation initiation and induced expression of *p63*, which may coordinate with BMP4 to accelerate epidermal specification by regulating accumulation of H3K27me3 [64]. Deletion of *p63* resulted in a significant decrease in signal of H3K27me3 mark [64]. We show that short telomeres can up-regulate *Fst* via reducing H3K27me3 at Fst promoter and decrease pSmad, resulting in declined expression of *p63*. These findings suggest a complex feedback mechanism between H3K27me3 and Fst-BMP4-P63. Fst, BMP4, P63, and H3K27me3 are key players in the orchestra that regulates epidermal differentiation.

Another interesting phenomenon for *Fst* promoter is its hypomethylated state. Additionally, methylation levels at subtelomeres of chromosome 13 where *Fst* gene is located are also drastically reduced in G4 *Terc*^*–/–*^ES cells, compared with those of WT ES cells. Coincidently, *Fst* and other subtelomeric genes such as *Tcstv1/3* in chromosome 13 are expressed at higher levels in G4 *Terc*^*–/–*^ES cells, but down-regulated in *Terc*-repaired G4 *Terc*^*–/–*^ES cells like WT cells (S8 Fig). DNA hypomethylation could lead to decreased levels of H3K27me3 in ordinarily unmethylated regions [18,65]. Our data suggests that H3K9me3-mediated gene silencing does not play a direct role in repressing *Fst*. We indeed find a global reduction of H3K9me3 in G4 *Terc*^–/–^ES cells in which H3K9me3 also shows reduced co-localization with telomeres. These data suggest that telomere shortening-induced reduction of H3K9me3 at telomeres/subtelomeres may have a general impact on gene de-repression instead of a direct impact on *Fst* gene.

Taken together, short or loss of telomere disrupts PRC2 function involving H3K27me3 and de-represses *Fst*. Elevated Fst inhibits pSmad/P63 signaling, leading to defective epidermal stem cell specification, stratification and differentiation. Rejuvenating telomere length can rescue these defects. We do not exclude the possibility that additional signaling pathways may also be involved in and/or cooperate with aberrant Fst/pSmad/P63 signaling in defective epidermal differentiation resulting from telomere dysfunction. Targeting Fst/pSmad/P63 pathway may have implications in ameliorating skin and hair degeneration associated with aging and telomere shortening.

## Materials and methods

### Ethics statement

All animal experiments were approved by the Institutional Animal Care and Use Committee at Nankai University (License number 20140006). All animal studies were carried out in strict accordance with the recommendations in the Guide for the Care and Use of Laboratory Animals of Nankai University. All efforts were made to minimize the number of animals used by the experimental design.

### Mice

Two-three month old *Terc* deficient (*Terc*^–/–^) mice and wild-type mice in C57Bl/6 background, and immunodeficient mice were used in this study. Mice were housed and cared for in a pathogen-free facility at Nankai University.

### ES cells and culture

*Terc*^–/–^ES cells were generated from *Terc* deficient mice and cultured as previously described [17]. N33 ES cell line was derived from wild-type mice, heterozygous (H1) ES cells from *Terc*^+/–^mice, and F19, F35, and A49 ES cell lines from G1, G3, G4 *Terc*^–/–^mice, respectively. These ES cells were maintained on mitomycin-C treated mouse embryonic fibroblasts as feeders in ES cell culture medium containing knockout Dulbecco’s modified Eagle medium (KO-DMEM) (Invitrogen) added with 20% fetal bovine serum (Hyclone), 1000 U/ml LIF, 0.1 mM β-mercaptoethanol, 1 mM L-glutamine, 0.1 mM non-essential amino acids, 100 units/ml penicillin and 100 μg/ml streptomycin.

### *Terc* repair in *Terc*^–/–^ES cell line by CRISPR/Cas9

pSpCas9(BB)-2A-Puro (PX459) was a gift from Feng Zhang (plasmid # 48139, Addgene). Guide RNAs were designed using the online design tool available at http://crispr.genome-engineering.org/. PX459 was digested with BbsI and then gel purified. Two pairs of oligos including targeting sequences were annealed and cloned into the BbsI-digested PX459 vector. The *Terc* donor sequences were obtained based on mouse genomic sequence and the information provided in the original paper [43]. The *Terc* donor vector contained *Terc* flanked by 5’(Left) and 3’(Right) homology arms. The DNA fragments are individually amplified by proper primers and then cloned into the vector with proper enzymes. G4 *Terc*^–/–^ES cell line A49 was transfected with two PX459 and *Terc* donor plasmids using lipofectamine 2000 transfection reagent (Invitrogen). Twenty-four hours later, 2 μg/ml puromycin was added into the culture medium for 7 days, clones were picked and the genomic DNA was extracted. PCR was performed with several pairs of primers to detect and obtain the genomic knock-in *Terc* repaired cell lines.

### *In vitro* differentiation of ES cells

ES cells are allowed to aggregate and form three-dimensional colonies known as embryoid bodies (EBs) [66]. Differentiation of ES cells was accomplished in a two-step process: (1) Embryoid body (EB) formation was obtained by using cell suspension and hanging drop method. Undifferentiated ES cells were trypsinized to obtain a single cell suspension, and EBs were formed in ES cell culture medium without LIF, in a definite number of cells in "hanging drops" for 4 days. (2) Then, EB were transferred to 24-well microwell plates with one EB per well. Daily microscopic observations were conducted to detect beating EBs. 10~15 EBs were transferred to 6-well microwell plates per well for protein, RNA, and DNA sample collection.

### Teratoma formation assay and histological analysis

Approximately 2×10^6^ ES cells with different telomere length were injected subcutaneously into dorsal flanks of immunodeficient mice. Four weeks after the injection, the mice were humanely sacrificed and the teratomas were surgically dissected from the mice. Samples were weighed, fixed in PBS containing 3.7% formaldehyde, and embedded in paraffin. Sections were stained with hematoxylin and eosin for histological examination.

### RNA extraction and qPCR

The total RNA was isolated from samples using TriZol (Invitrogen) or RNeasy mini kit (Qiagen) according to the manufacturer's protocol. The purity and concentration of RNA were checked using Nanodrop technology (Agilent). 2μg RNA was subjected to cDNA synthesis using M-MLV Reverse Transcriptase (Invitrogen). Quantitative real-time PCR reactions were set up in duplicate with the FastStart Universal SYBR Green Master (ROX) (Roche) and run on the iCycler iQ5 2.0 Standard Edition Optical System (Bio-Rad). Each sample was repeated at least twice and analyzed with Gapdh served as the internal control. Quantification of gene expression was based on the Ct (Cycle threshold) value. Melting curve analysis and electrophoresis were performed to control PCR products specificities and exclude nonspecific amplification. PCR Primers, designed using Primer5 and Gene Runner software, are listed in **S1 Table**.

### Western blot

Cells were collected and washed with cold phosphate buffered saline (PBS), then resuspended in cell lysis buffer containing 50 mM Tris (pH 7.4), 150 mM NaCl, 1 mM EDTA, 1 mM EGTA, 1 mM NaF, 20 mM Na_4_P_2_O_7_, 1 mM Na_3_VO_4_, 1%Triton X-100, 10% glycerol, 0.25% deoxycholate and 0.1% SDS. 20 μg of proteins were separated on 10% SDS-polyacrylamide gels and transferred to polyvinylidene difluoride (PVDF, Millipore) membrane. Nonspecific binding was blocked by incubation in 5% nonfat dry milk in TBST at room temperature. Blots were then probed overnight at 4°C with primary antibodies against K14 (ab7800, Abcam), P63 (ab124762, Abcam), H3 (ab1791, Abcam), H3K4me3 (ab1012, Abcam), H3K9me3 (07–442, Millipore), H3K27me3 (07–449, Millipore), Smad1 (#9743, CST), pSmad1/5/8 (#9511, CST), pSmad2/3(#8828, CST), Smad2/3(#5678, CST), Fst (ab64490, Abcam), Dnmt3a (ab13888, Abcam), Dnmt3b (ab13604, Abcam), or β-actin (sc1616R, Santa Cruz), washed and incubated for 2 h with secondary antibodies HRP conjugated donkey anti-Rabbit IgG (NA934v GE Healthcare) or goat anti-mouse IgG (H+L) (ZB2305). Protein bands were detected using ECL western blotting detection reagents (WBKLS0100 Millipore). The band intensity was measured by software ImageJ and normalized to the intensity of β-actin. The relative expression level was calculated from the results of at least three independent experiments or samples and presented as mean ± SEM [67].

### Telomere measurement by qPCR

Cells were washed in PBS and stored at -20°C until subsequent DNA extraction. Genome DNA was prepared using DNeasy Blood & Tissue Kit (Qiagen, Valencia, CA). Average telomere length was measured from total genomic DNA using a real-time PCR assay, as previously described [68], but modified for measurement of mouse telomere [69]. PCR reactions were performed on the iCycler iQ5 2.0 Standard Edition Optical System (Bio-Rad, Hercules, CA), using telomeric primers, primers for the reference control gene (mouse 36B4 single copy gene) and PCR settings as previously described [70]. For each PCR reaction, a standard curve was made by serial dilutions of known amounts of DNA. The telomere signal was normalized to the signal from the single copy gene to generate a T/S ratio indicative of relative telomere length. Equal amounts of DNA (20 ng) were used for each reaction. The primers for telomere measurement by qPCR are listed in **S2 Table**.

### Telomere quantitative fluorescence *in situ* hybridization (QFISH)

Telomere length and function (telomere integrity and chromosome stability) were estimated by telomere quantitative FISH [17,43]. Briefly, cells were incubated with 0.5 μg/ml nocodazole for 1.5 h to enrich cells at metaphases. Chromosome spreads were made by standard method. Metaphase-enriched cells were exposed to hypotonic treatment with 75 mM KCl solution, fixed with methanol: glacial acetic acid (3:1) and spread onto clean slides. Telomeres were denatured at 80°C for 3 min and hybridized with FITC-labeled telomere (CCCTAA) peptide nucleic acid (PNA) probe (0.5 μg/ml) (Panagene, Korea). Chromosomes were stained with 0.5 μg/ml DAPI. Fluorescence from chromosomes and telomeres was digitally imaged on a Zeiss microscope with fluorescein isothiocyanate (FITC)/DAPI filters, using AxioCam and AxioVision software 4.6. Telomere length shown as telomere fluorescence intensity was integrated using the TFL-TELO program (a gift kindly provided by Peter Lansdorp).

### Telomerase activity by TRAP assay

Telomerase activity was measured by the Stretch PCR method according to the manufacturer’s instruction using TeloChaser Telomerase assay kit (T0001, MD Biotechnology). Briefly, about 2.5 × 10^4^ cells from each sample were lysed. Lysis buffer served as negative controls. PCR products of cell lysates were separated on non-denaturing TBE-based 12% polyacrylamide gel electrophoresis and visualized by ethidium bromide staining.

### Telomere Restriction Fragment (TRF) measurement

TRF analysis was performed using a commercial kit (TeloTAGGG Telomere Length Assay, catalog no. 12209136001, Roche Life Science). Cells were pretreated with RNaseA and Proteinase K (PCR Grade, 03115879001, Roche Life Science), followed by extraction using phenol: chloroform: isoamyl alcohol, digested with MboI (R0147, NEB) at 37 °C overnight and electrophoresed through 1% agarose gels in 0.5 × TBE at 14 °C using a CHEF Mapper pulsed field electrophoresis system (Bio-rad). Auto algorithm was used to separate DNA samples with a size range from 5 to 150 kb. The gel was blotted and probed using reagents in the kit.

### Immunofluorescence microscopy

Tail or back skin tissues obtained from wild-type (WT) or G3 *Terc* deficient mice, or teratomas were fixed overnight in 3.7% paraformaldehyde at 4°C, dehydrated through graded alcohols and xylene, and embedded in paraffin. After deparaffinizing, rehydrating and washing in PBS, sections were incubated with 3% H_2_O_2_ for 10 min at room temperature to block endogenous peroxidase, subjected to high pressure antigen recovery sequentially in 0.01% citrate buffer for 3 min, blocked with 5% goat serum in PBS for 2 h at room temperature, and then incubated with the primary antibodies against K14 (ab7800, Abcam), Fst (ab64490, Abcam) or P63 (ab124762, Abcam) overnight at 4°C, washed and incubated for 2 h with appropriate fluorescence-conjugated secondary antibodies (Goat anti Mouse IgG (H+L), FITC, 115-095-003, Jackson; Goat anti Rabbit IgG (H+L), Alexa Fluor 594, 111-585-003, Jackson). For immunostaining of ES cells and *in vitro* differentiated cells, they were washed twice in PBS, then fixed in freshly prepared 3.7% paraformaldehyde in PBS (pH 7.4), permeabilized in 0.1% Triton X-100 (Sigma–Aldrich, Saint Louis, MO) in blocking solution (3% goat serum plus 0.5% BSA in PBS) for 30 min, washed and left in blocking solution for 1 h. Cells were then incubated overnight at 4°C with primary antibodies and then secondary antibodies as described above. Blocking solution without the primary antibody served as negative control. Nuclei were counterstained with 0.5 μg/ml Hoechst 33342 in Vectashield mounting medium. Fluorescence was imaged using a Zeiss fluorescence microscope (Axio Imager Z1) and using the same exposure time for each group. ImageJ software (https://imagej.net/) was used for relative quantity measurement of fluorescence intensity. Region-of-interest (ROI) tool was used to select the cell or background, and the fluorescence intensity of ROIs achieved. Background with the same threshold was subtracted for each image.

### Immunofluorescence-telomere FISH (IF-FISH)

IF-FISH was performed based on an established protocol [71]. Briefly, immunostaining of the cells was performed as described above. After washing the excess of secondary antibody with PBS, cells were fixed in 4% formaldehyde for 2 min, dehydrated with ethanol, and incubated with FITC-telomeric PNA probe as described earlier for telomere QFISH. Fluorescence was imaged using the Zeiss fluorescence microscope.

### Bisulfite genomic sequencing

DNA methylation by bisulfite sequencing Genomic DNA was extracted from cells using DNeasy & Blood Tissue Kit (Qiagen) according to the manufacturer’s instructions. Bisulfite treatment of DNA was performed with the EpiTect Bisulfite Kit (Qiagen). Bisulfite converted DNA was amplified by seminested PCR, using HS EX Taq DNA Polymerase (Takara). Primer sequences are detailed in **S3 Table**. PCR products were recovered from stained gels (EasyPure Quick Gel Extraction Kit,Transgen), cloned into a pEASY-T1 Simple Cloning vector (Transgen) and then sequenced.

### Overexpression of *Fst* or *p63*

The plasmid pEASY-T1-*Fst*- overexpression (OE) and pEASY-T1-*p63*-OE were constructed by amplification of *Fst* or *p63* cDNA by PCR and cloning it into pEASY-T1 simple cloning vector (TransGen). Following digestion with XhoI and NotI, *Fst* or *p63* sequences were inserted into Plch37 plasmid. Then the recombinant plasmids were transfected into J1 ES cells or MEF. At 48 h after transfection with 2 μg plasmid using lipofectamine 2000 (Invitrogen), cells were collected for protein and RNA extraction. For obtaining stably transfected cell lines with *Fst* overexpression, cells were transfected with 2 μg plasmid using lipofectamine 2000 (Invitrogen) and selected by 1.5 μg/ml puromycin for 7–10 days, and clones were picked.

### *Fst* RNAi

shRNA sequences were synthesized (**S4 Table**), and cloned into pSIREN-RetroQ, according to manufacturer’s instructions. The shRNAs without sequence homology to mouse genes served as a negative control. The RNAi retrovirus was packaged using Plat-E cells and then infected cells during differentiation.

### ChIP-qPCR assay

ChIP-qPCR analysis was performed as described previously [72], with slight modification. Briefly, 5 × 10^7^ cells were fixed with 1% paraformaldehyde, lysed, and sonicated to achieve the majority of DNA fragments with 100–1000 bp. DNA fragments were then enriched by immunoprecipitation with 5 μg H3K9me3 antibody (ab8898, Abcam), 7 μg H3K9Ac antibody (ab4441, Abcam), 5 μg Dnmt3b antibody (ab13604, Abcam), 5 μg H3K9me2 antibody (ab1220, Abcam) or 5 μg H3K27me3 (ab6002, Abcam). The eluted protein:DNA complex was reverse-crosslinked at 65 °C overnight. DNA was recovered after proteinase and RNase A treatment. Real-time PCR was performed to compare the histone modification at the *Fst* promoter region using primers provided in **S5 Table**. Normal rabbit IgG (#2729S, Cell Signaling) or Mouse (G3A1) mAb IgG1 Isotype Control (5415S, Cell Signaling) served as negative control.

### Genome-wide gene expression by microarray analysis

Microarray was performed by CapitalBio Corporation (Beijing, China) using Affymetrix 430 2.0 oligonucleotide mouse arrays designed from GenBank, dbEST, and RefSeq sequences based on the UniGene database. The analysis was carried out based on the software and method provided by CapitalBio (http://www.capitalbio.com). Only probe sets showing at least 1.8-fold change were retained in the final list. The detection call indicates whether a transcript was reliably detected (P, Present) or not (A, Absent). We performed hierarchical clustering with the differentially expressed genes using cluster software (version 3) and by applied mean centering and normalization of genes and arrays prior to average linkage clustering.

### Luciferase reporter assay

The *Fst* promoter (~2000bp) was cloned into pGL3-basic vector, following digestion with XhoI and HindIII. 2×10^5^ ES cells per 12 well were transfected with 1 μg pGL3-basic vector containing *Fst* promoter and 10 ng pRL-SV40 vector as control using lipofectamine 2000 (Invitrogen) according to manufacturer’s instruction. 24 hours after transfection, ES cells were lysed with 1×PLB (positive lysis buffer, Promega), shaken for 15 min, and then centrifuged at 13000 rpm for 10 min at 4°C. The supernatants were collected and analyzed for luciferase activity by dual reporter assay according to manufacturer’s instructions.

### Chromosome XMP13 FISH and telomere FISH

XMP13 probe (D-1413, Metasystems) specific for mouse Chromosome 13 was used for chromosome identification. FISH on chromosome spread was performed according to manufacturer’s instructions. The probe was added and coverslip placed, sealed with rubber cement, denatured by heating slide at 75°C for 2 min, and incubated in humidified chamber at 37°C overnight. Slides were washed and stained with 0.5 μg/ml DAPI in VectaShield antifade medium. Digital images were captured using a CCD camera on a Zeiss Imager Z2 microscope. The coordinates of the chromosome were recorded with the venire scale along the top and side of the microscope stage. The slides were washed and performed with telomere FISH as described above. After staining with DAPI again, fluorescence from chromosomes and telomeres was digitally imaged using the same microscope according to the recorded coordinates. The telomeres of chromosome 13 were revealed by comparison of the images from the same coordinates.

### Statistical analysis

The data from multiple groups were analyzed by ANOVA, and means were compared by Fisher’s protected least significant difference (PLSD) using the StatView software from SAS Institute. T-test was used to analyze statistical significance of the two-paired groups. Significant differences were defined as p < 0.05, 0.01, or lower.

## Supporting information

S1 Fig *In vitro* differentiation of ES cells with various telomere lengths. (A) Morphology of colonies of ES cells (WT, *Terc*^+/–^, G1, G3, and G4 *Terc*^–/–^ES cells), embryoid body (EB) at day 4, and differentiated cells by day 8 and day 15. (B&C) Relative expression level of pluripotent marker genes *Oct4* (B) and *Nanog* (C) at indicated time points of differentiation. Bars = Mean ± SEM (n = 3). (D) Methylation level of *Nanog* promoter analyzed at day 0 and day 15 of differentiation in WT and G4 *Terc*^–/–^ES cells. Genomic DNA was treated with bisulfite, followed by PCR amplification and sequencing. Circles, CpG sites within the regions analyzed; filled circles, methylated cytosines indicated by percentages underneath; open circles, unmethylated cytosines. (E&F) Relative mRNA levels by qPCR analysis of epidermal stem cell marker *p63* (E), and epidermis basal layer markers *K14*, *K5*, *K4*, and *K1* (F) at day 0, day 8, and day 15 of *in vitro* differentiation. Bars = Mean ± SEM (n = 3). *, p<0.05; **, p<0.01, compared to WT ES cells at the same time point. ES cells, embryonic stem cells; WT, wild type; *K14*, *Keratin 14; K5*, *keratin 5*; *K4*, *keratin 4*; *K1*, *keratin 1*; EB, embryoid body.
(TIF)

S2 Fig Short telomeres impair epidermal differentiation *in vivo*. **(**A) Epidermal differentiation in teratomas from WT and G4 *Terc*^–/–^ES cells as shown by immunofluorescence (IF) of P63 and K14. Scale bar = 50 μm. (B) Representative images showing skin (tail) of WT and G3 *Terc*^–/–^mice revealed by immunofluorescence of K14 and P63 and histology by H&E staining. Scale bar = 50 μm. (C) Thickness of skin epidermis in WT and G3 *Terc*^–/–^mice estimated from H&E histology. *, p<0.05.
(JPG)

S3 Fig Co-immunofluorescence of P63 or K14 with Fst expression. (A) Representative immunofluorescence images showing co-staining of K14 (green) with Fst (red) in sections of mouse back skin. Scale bar = 20 μm. (B) Representative immunofluorescence images showing co-staining of K14 with Fst in the sections of mouse skin epidermis. WT mouse skin displays many hair follicles underneath and G3 *Terc*^–/–^mouse skin shows fewer and smaller hair follicles. Scale bar = 25 μm.
(TIF)

S4 Fig Genome-wide gene expression profile showing differential gene expression in WT cells with long telomeres and G4 *Terc*^–/–^knockout (KO) cells with shortest telomeres. (A) Heatmap illustrating relative expression pattern of G4 *Terc*^–/–^cells compared to WT cells in duplicates. The genes with changes > = 1.8-fold between two groups were chosen for heatmap. The number of differentially expressed genes increased during the differentiation. (B) Heatmap highlighting relative expression pattern of genes related to DNA methylation, pluripotency, BMP/TGF-β signaling pathway and epidermis in G4 *Terc*^–/–^cells compared to WT cells in duplicates. During differentiation, WT ES cells exhibit significant reduction in expression of pluripotency genes, but G4 *Terc*^–/–^ES cells still maintain relatively high expression levels of pluripotency genes. On the contrary, BMP/TGF-β signaling genes are expressed at higher levels during differentiation of WT (wild type) ES cells, but at reduced levels in G4 *Terc*^–/–^ES cells. (C) Relative expression levels of genes related to BMP4/Smad1 pathway analyzed by qPCR in ES cells with various telomere lengths. BMP, bone morphogenetic protein.
(TIF)

S5 Fig Comparison of DNA methylation levels in ES cells and following differentiation. (A) Protein levels of both Dnmt3a and Dnmt3b are lower in G3/G4 *Terc*^–/–^ES cells than in WT ES cells by Western blot. However, Dnmt3b levels are higher and Dnmt3a lower in G3/G4 *Terc*^–/–^cells than in WT cells by day 15 of differentiation. β-actin served as loading control. (B) Real-time PCR based ChIP analysis of Dnmt3b abundance at *Fst* promoter region in WT and G4 *Terc*^–/–^ES cells. Bars = Mean ± SEM (n = 4). (C) Methylation status of *Fst* in WT ES cells, G4 *Terc*^–/–^ES cells, and MEF. (D) Methylation level of subtelomere region of Chr13 in ES cells and at day 15 following differentiation. Genomic DNA was treated with bisulfite, followed by PCR amplification and sequencing. Circles, CpG sites within the regions analyzed; filled circles, methylated cytosines indicated by percentages underneath; open circles, unmethylated cytosines. MEF, mouse embryonic fibroblasts cells.
(TIF)

S6 Fig Histone levels in ES cells with various telomere lengths and following differentiation. (A) Histone levels by Western blot analysis of WT, *Terc*^+/–^, G1, G3, and G4 *Terc*^–/–^ES cells prior to differentiation (day 0) and at day 8, day 15 of differentiation. Histone H3 served as loading control. (B) Immunofluorescence and co-localization of H3K9me3 distribution and foci and telomere FISH in WT and G4 *Terc*^–/–^ES cells at day 0 or at day 15 of differentiation. Relative H3K9me3 immunofluorescence intensity was estimated by Image J software. ***, P<0.001.
(JPG)

S7 Fig Regulation of *Fst* by Ezh1, Ezh2 and H3K27me3. (A) *Fst* promoter activity is higher in G4 *Terc*^–/–^than in WT ES cells. Mean ± SEM (n = 3). **, p<0.01. (B) Relative expression levels of genes associated with PRC2 and H3K27me3 by qPCR analysis. (C) Expression levels by qPCR of *Ezh1* and *Ezh2* in WT, G4 *Terc*^–/–^and G4 *Terc*-repaired ES cells. Bars = Mean ± SEM (n = 3). *, p<0.05; **, p<0.01, compared with WT ES cells. (D) ChIP-qPCR analysis of H3K27me3 abundance at *Fst* promoter region in WT, G4 *Terc*^–/–^and *Terc* repaired ES cells, showing decreased level of H3K27me3 at Fst promoter in cells with short telomere. Mean ± SEM (n = 3). β-actin served as control. *, p<0.05; **, p<0.01. (E&F) Immunofluorescence of Nanog and Oct4 at day 0 (E) and day 15 (F) of differentiation in WT, G4 *Terc*^–/–^and G4 *Terc*-repaired cells. Scale bar = 20 μm.
(TIF)

S8 Fig Heatmap illustrating relative expression pattern of representative genes located between Fst and telomere in WT ES cells, G4 *Terc*^–/–^knockout ES cells and *Terc* repaired G4 *Terc*^–/–^ES cells. The genes near the end of long arm of chromosome 13 with expression levels with FPKM more than 1 by RNA-seq are shown.
(TIF)

S1 Table Primers for quantitative real-time PCR analysis. (DOCX)

S2 Table Primers used for telomere length measurement by qPCR. (DOCX)

S3 Table Primers for methylation analysis. (DOCX)

S4 Table shRNA sequences targeting to *Fst*. (DOCX)

S5 Table Primers for ChIP-qPCR. (DOCX)
