# Supplementary figures and images for: Telomere dysfunction impairs epidermal stem cell specification and differentiation by disrupting BMP/pSmad/P63 signaling
Source: PLoS Genet. 2019 Sep 13;15(9):e1008368. doi: 10.1371/journal.pgen.1008368 (PMC6760834; doi:10.1371/journal.pgen.1008368)

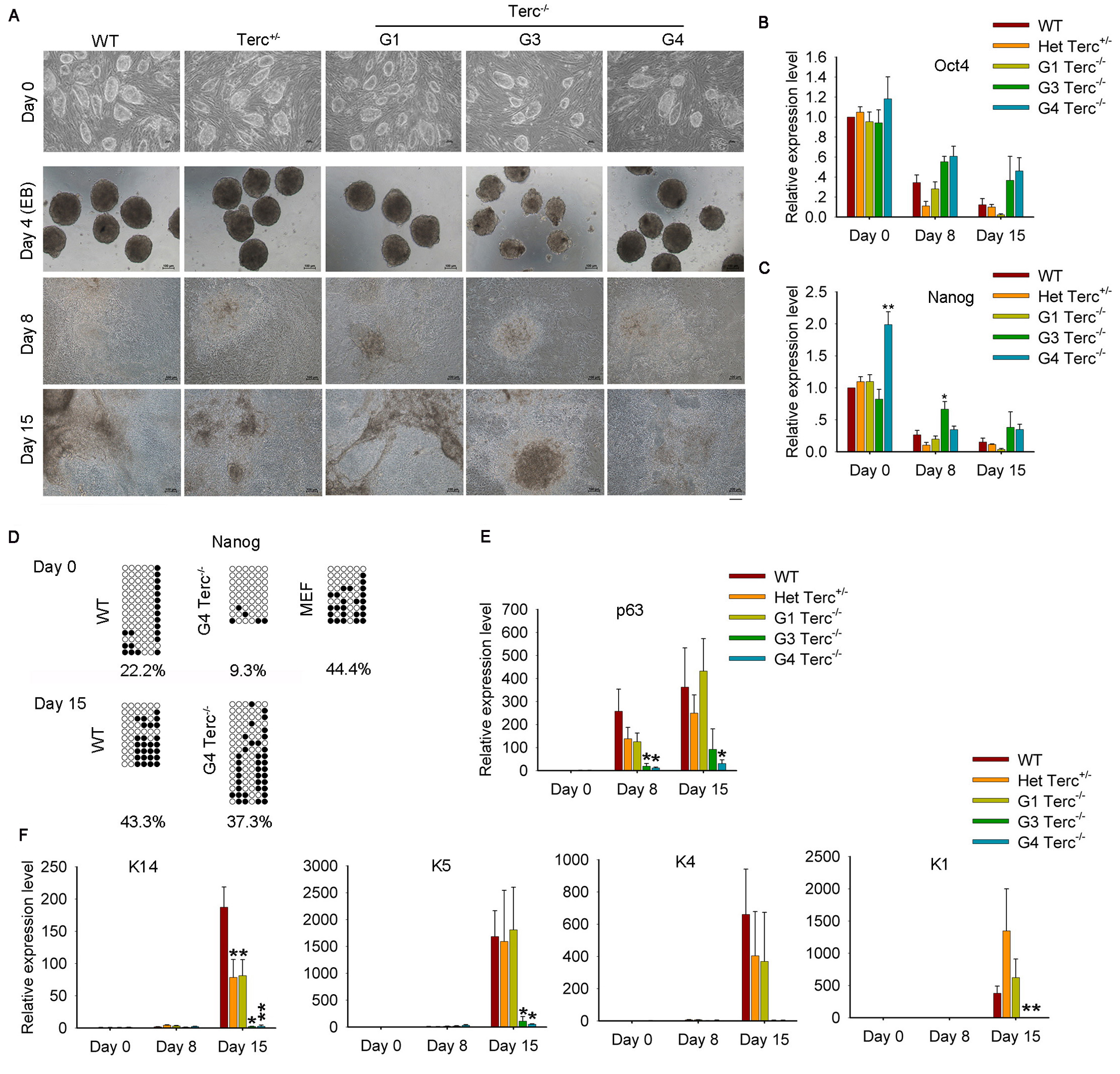

Supplement: S1 Fig — (A) Morphology of colonies of ES cells (WT, Terc+/–, G1, G3, and G4 Terc–/–ES cells), embryoid body (EB) at day 4, and differentiated cells by day 8 and day 15. (B&C) Relative expression level of pluripotent marker genes Oct4 (B) and Nanog (C) at indicated time points of differentiation. Bars = Mean ± SEM (n = 3). (D) Methylation level of Nanog promoter analyzed at day 0 and day 15 of differentiation in WT and G4 Terc–/–ES cells. Genomic DNA was treated with bisulfite, followed by PCR amplification and sequencing. Circles, CpG sites within the regions analyzed; filled circles, methylated cytosines indicated by percentages underneath; open circles, unmethylated cytosines. (E&F) Relative mRNA levels by qPCR analysis of epidermal stem cell marker p63 (E), and epidermis basal layer markers K14, K5, K4, and K1 (F) at day 0, day 8, and day 15 of in vitro differentiation. Bars = Mean ± SEM (n = 3). *, p<0.05; **, p<0.01, compared to WT ES cells at the same time point. ES cells, embryonic stem cells; WT, wild type; K14, Keratin 14; K5, keratin 5; K4, keratin 4; K1, keratin 1; EB, embryoid body. (TIF) [file pgen.1008368.s001.tif]

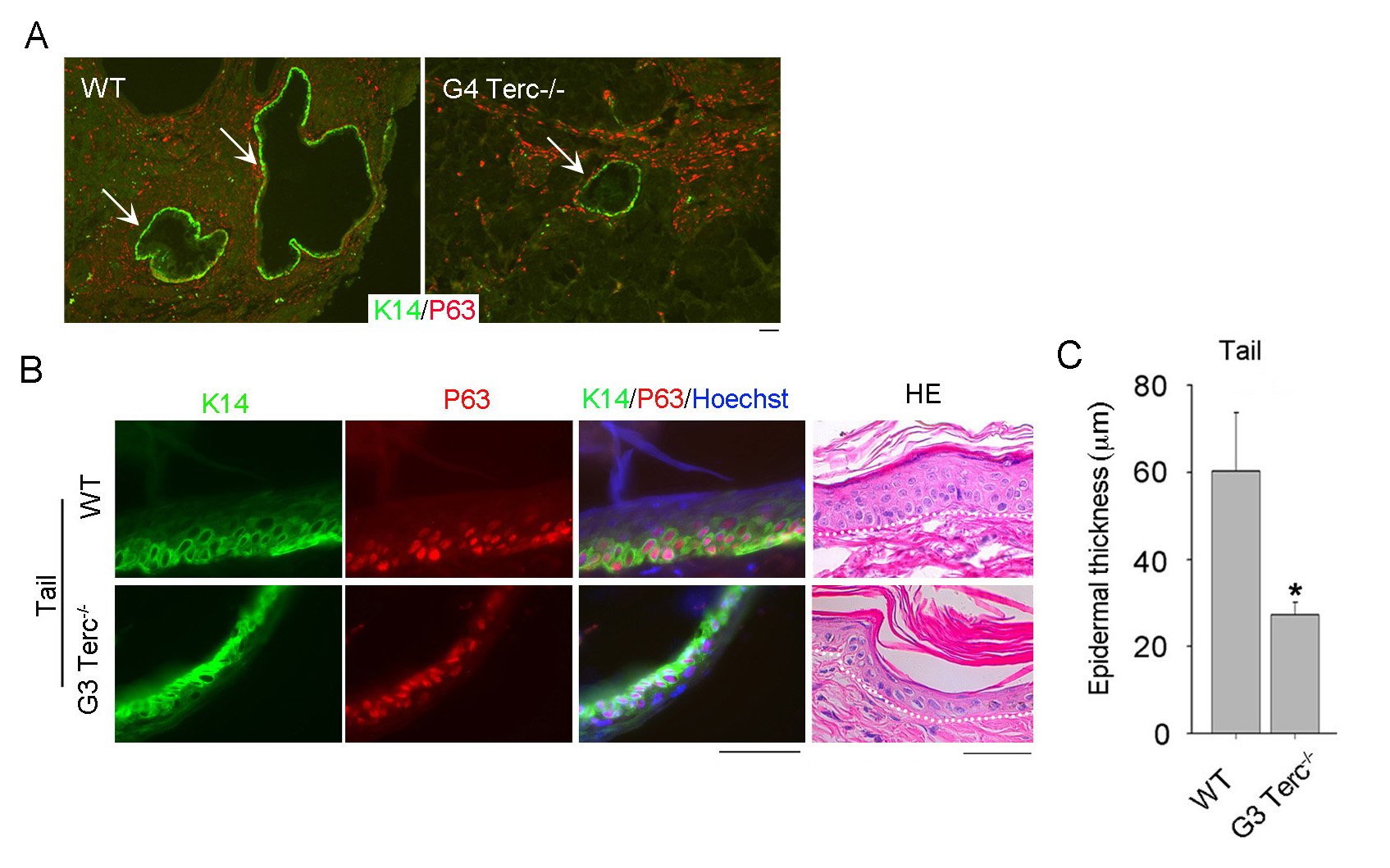

Supplement: S2 Fig — (A) Epidermal differentiation in teratomas from WT and G4 Terc–/–ES cells as shown by immunofluorescence (IF) of P63 and K14. Scale bar = 50 μm. (B) Representative images showing skin (tail) of WT and G3 Terc–/–mice revealed by immunofluorescence of K14 and P63 and histology by H&E staining. Scale bar = 50 μm. (C) Thickness of skin epidermis in WT and G3 Terc–/–mice estimated from H&E histology. *, p<0.05. (JPG) [file pgen.1008368.s002.jpg]

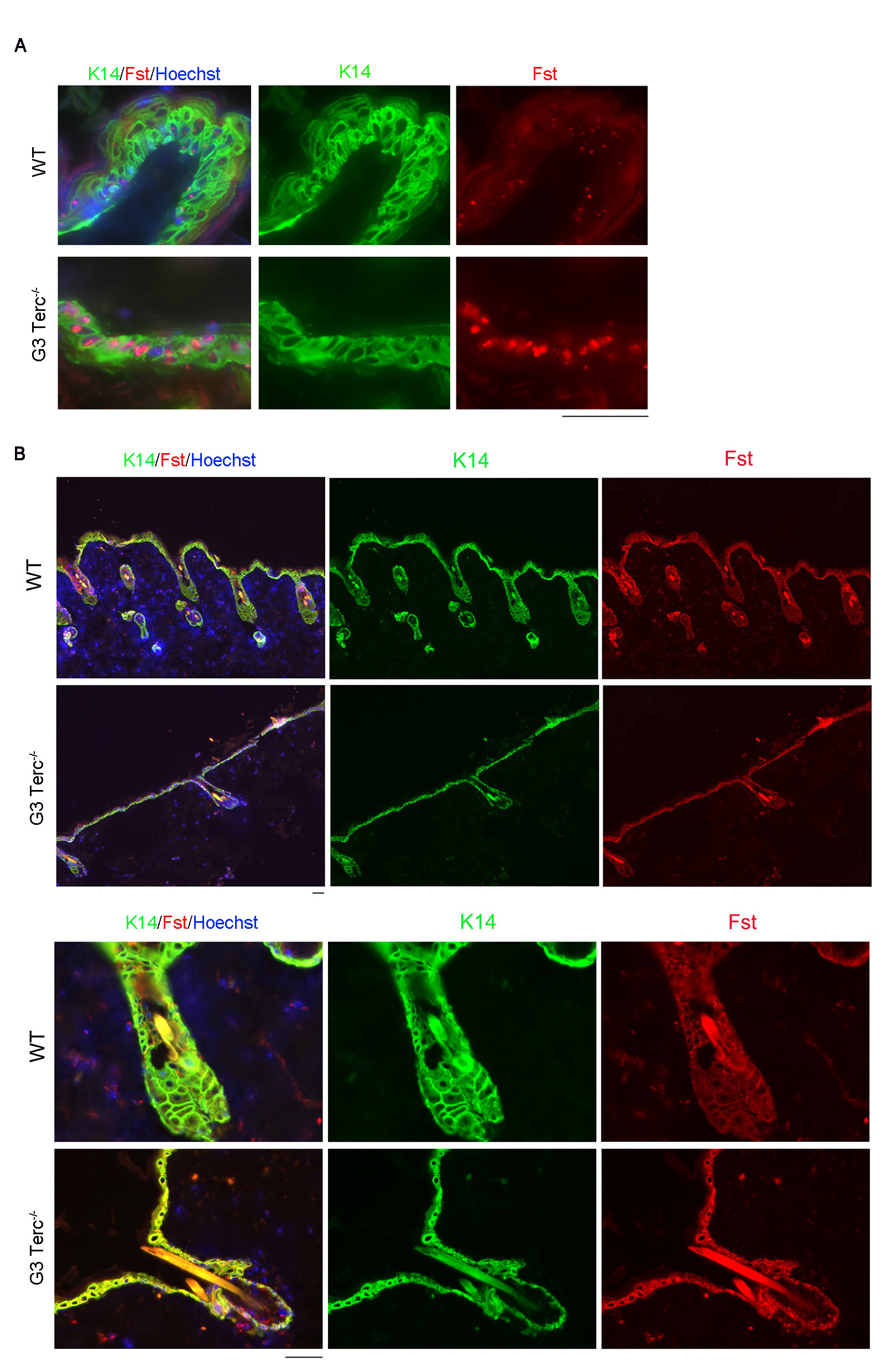

Supplement: S3 Fig — (A) Representative immunofluorescence images showing co-staining of K14 (green) with Fst (red) in sections of mouse back skin. Scale bar = 20 μm. (B) Representative immunofluorescence images showing co-staining of K14 with Fst in the sections of mouse skin epidermis. WT mouse skin displays many hair follicles underneath and G3 Terc–/–mouse skin shows fewer and smaller hair follicles. Scale bar = 25 μm. (TIF) [file pgen.1008368.s003.tif]

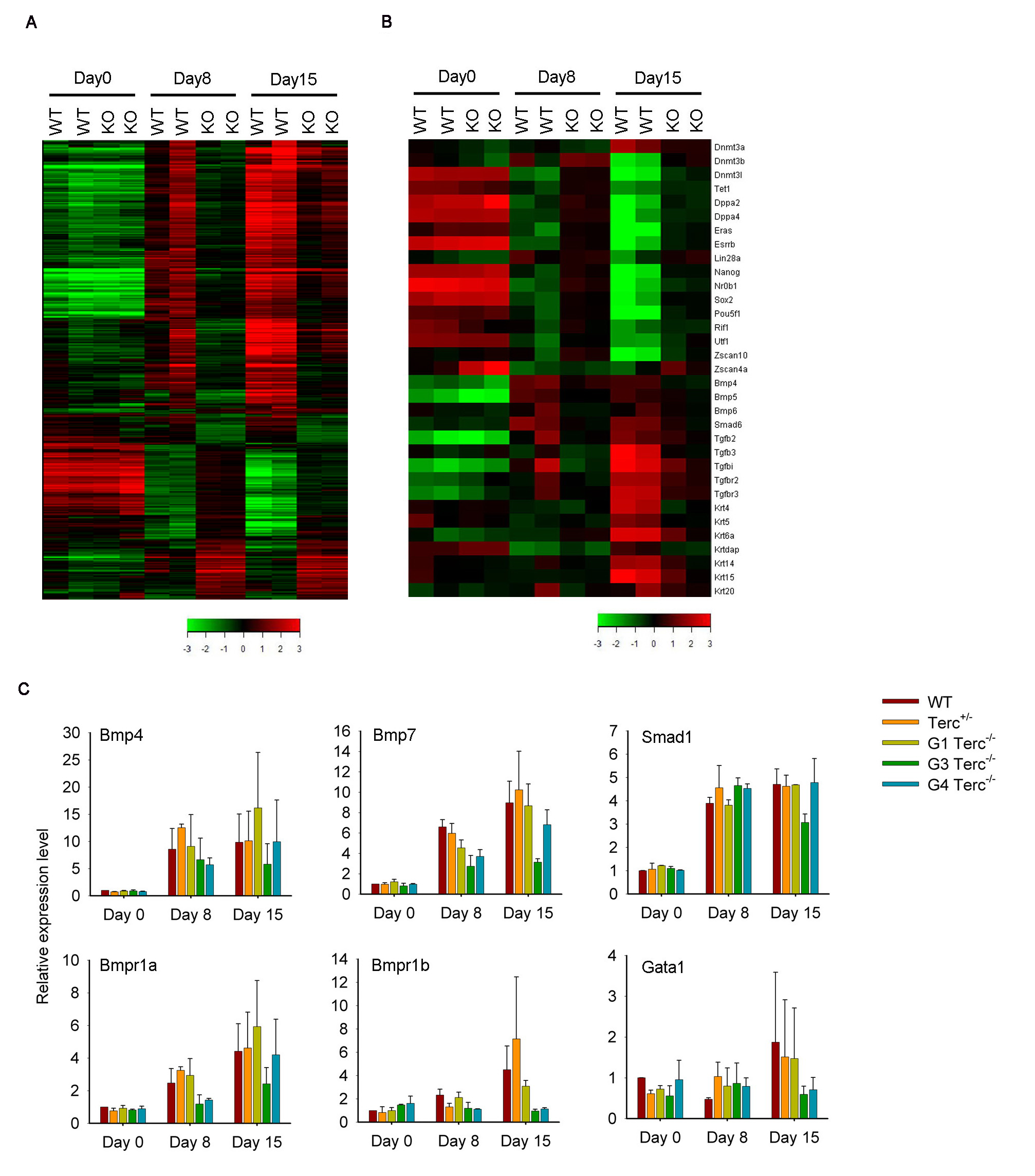

Supplement: S4 Fig — (A) Heatmap illustrating relative expression pattern of G4 Terc–/–cells compared to WT cells in duplicates. The genes with changes > = 1.8-fold between two groups were chosen for heatmap. The number of differentially expressed genes increased during the differentiation. (B) Heatmap highlighting relative expression pattern of genes related to DNA methylation, pluripotency, BMP/TGF-β signaling pathway and epidermis in G4 Terc–/–cells compared to WT cells in duplicates. During differentiation, WT ES cells exhibit significant reduction in expression of pluripotency genes, but G4 Terc–/–ES cells still maintain relatively high expression levels of pluripotency genes. On the contrary, BMP/TGF-β signaling genes are expressed at higher levels during differentiation of WT (wild type) ES cells, but at reduced levels in G4 Terc–/–ES cells. (C) Relative expression levels of genes related to BMP4/Smad1 pathway analyzed by qPCR in ES cells with various telomere lengths. BMP, bone morphogenetic protein. (TIF) [file pgen.1008368.s004.tif]

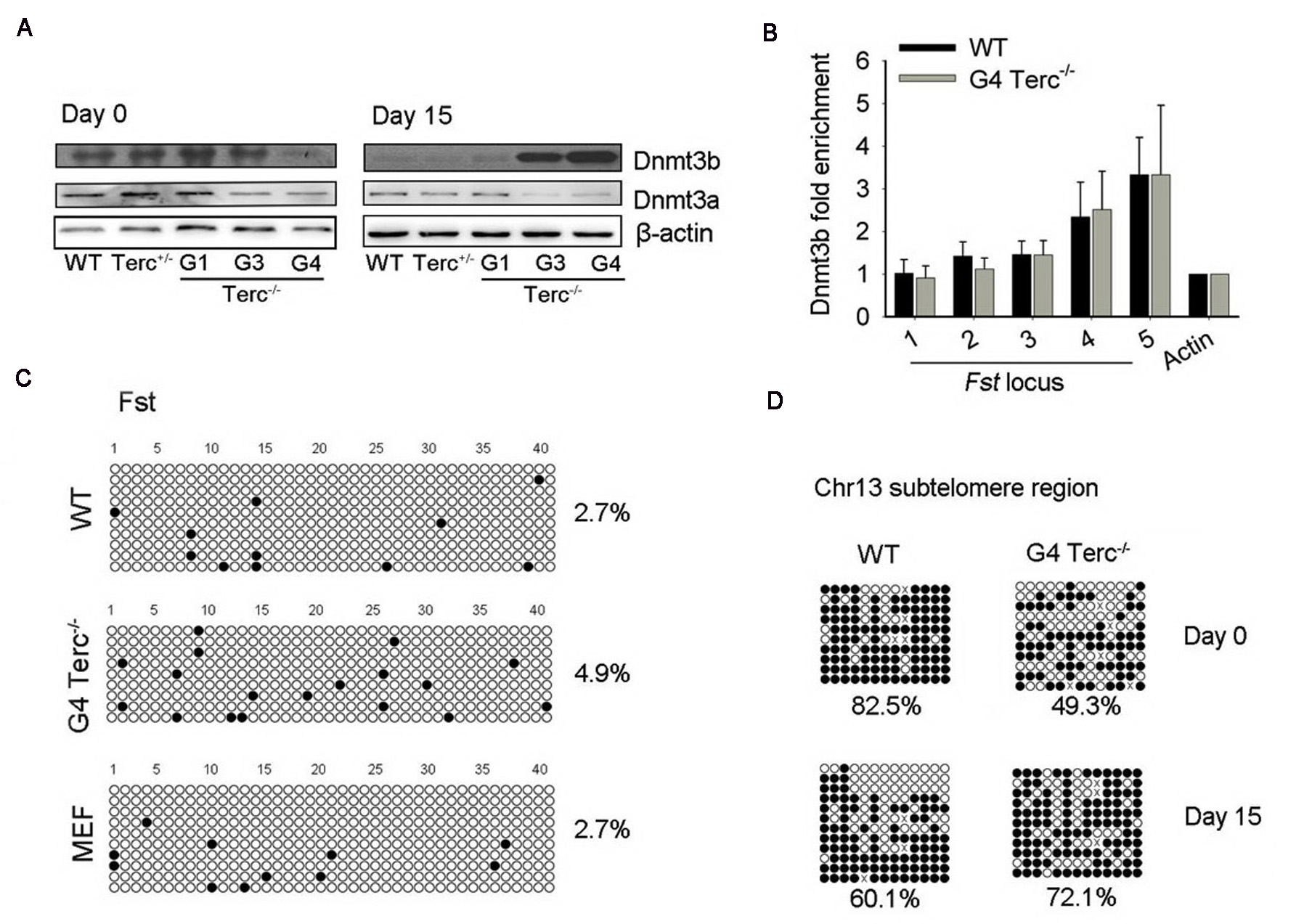

Supplement: S5 Fig — (A) Protein levels of both Dnmt3a and Dnmt3b are lower in G3/G4 Terc–/–ES cells than in WT ES cells by Western blot. However, Dnmt3b levels are higher and Dnmt3a lower in G3/G4 Terc–/–cells than in WT cells by day 15 of differentiation. β-actin served as loading control. (B) Real-time PCR based ChIP analysis of Dnmt3b abundance at Fst promoter region in WT and G4 Terc–/–ES cells. Bars = Mean ± SEM (n = 4). (C) Methylation status of Fst in WT ES cells, G4 Terc–/–ES cells, and MEF. (D) Methylation level of subtelomere region of Chr13 in ES cells and at day 15 following differentiation. Genomic DNA was treated with bisulfite, followed by PCR amplification and sequencing. Circles, CpG sites within the regions analyzed; filled circles, methylated cytosines indicated by percentages underneath; open circles, unmethylated cytosines. MEF, mouse embryonic fibroblasts cells. (TIF) [file pgen.1008368.s005.tif]

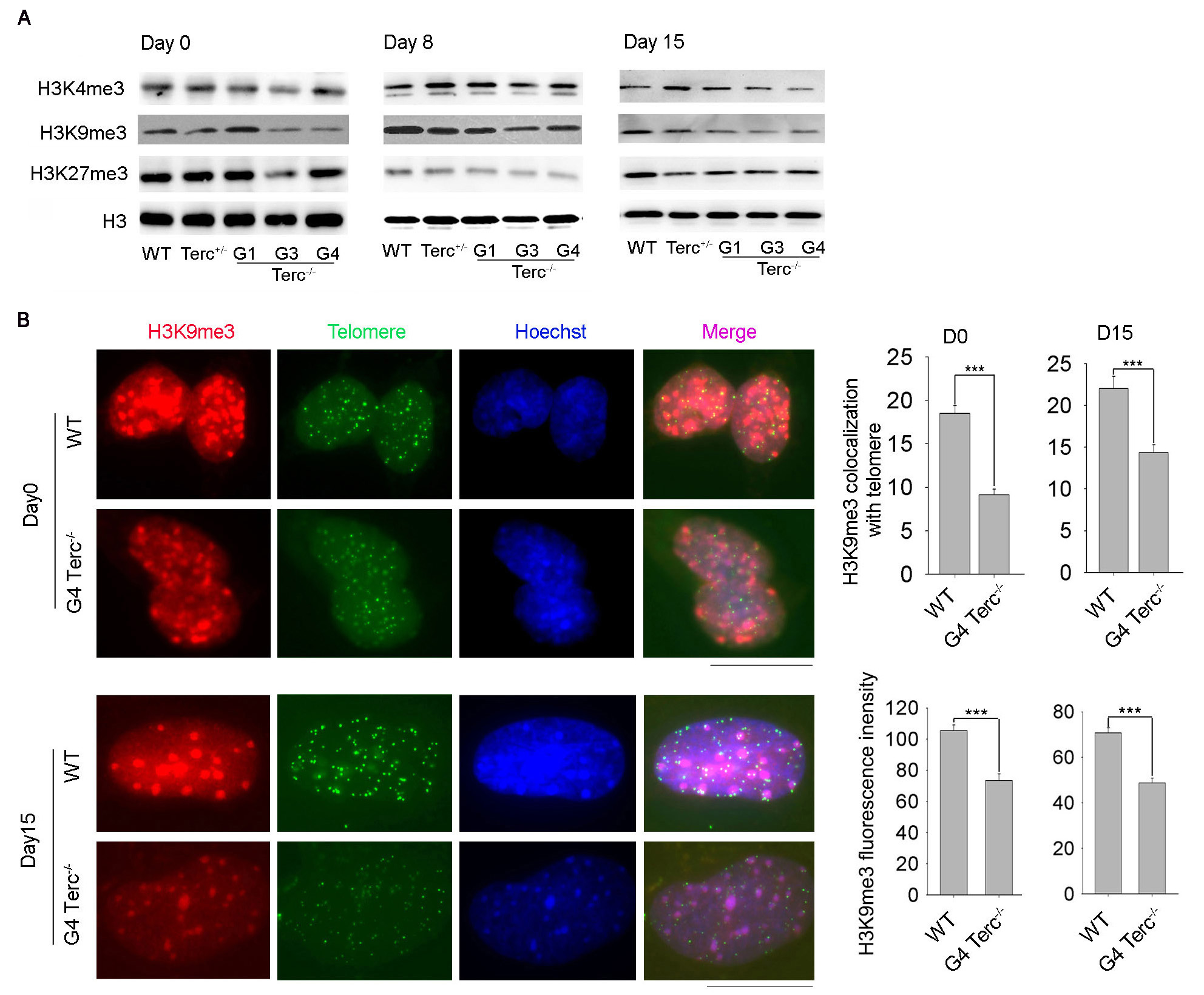

Supplement: S6 Fig — (A) Histone levels by Western blot analysis of WT, Terc+/–, G1, G3, and G4 Terc–/–ES cells prior to differentiation (day 0) and at day 8, day 15 of differentiation. Histone H3 served as loading control. (B) Immunofluorescence and co-localization of H3K9me3 distribution and foci and telomere FISH in WT and G4 Terc–/–ES cells at day 0 or at day 15 of differentiation. Relative H3K9me3 immunofluorescence intensity was estimated by Image J software. ***, P<0.001. (JPG) [file pgen.1008368.s006.jpg]

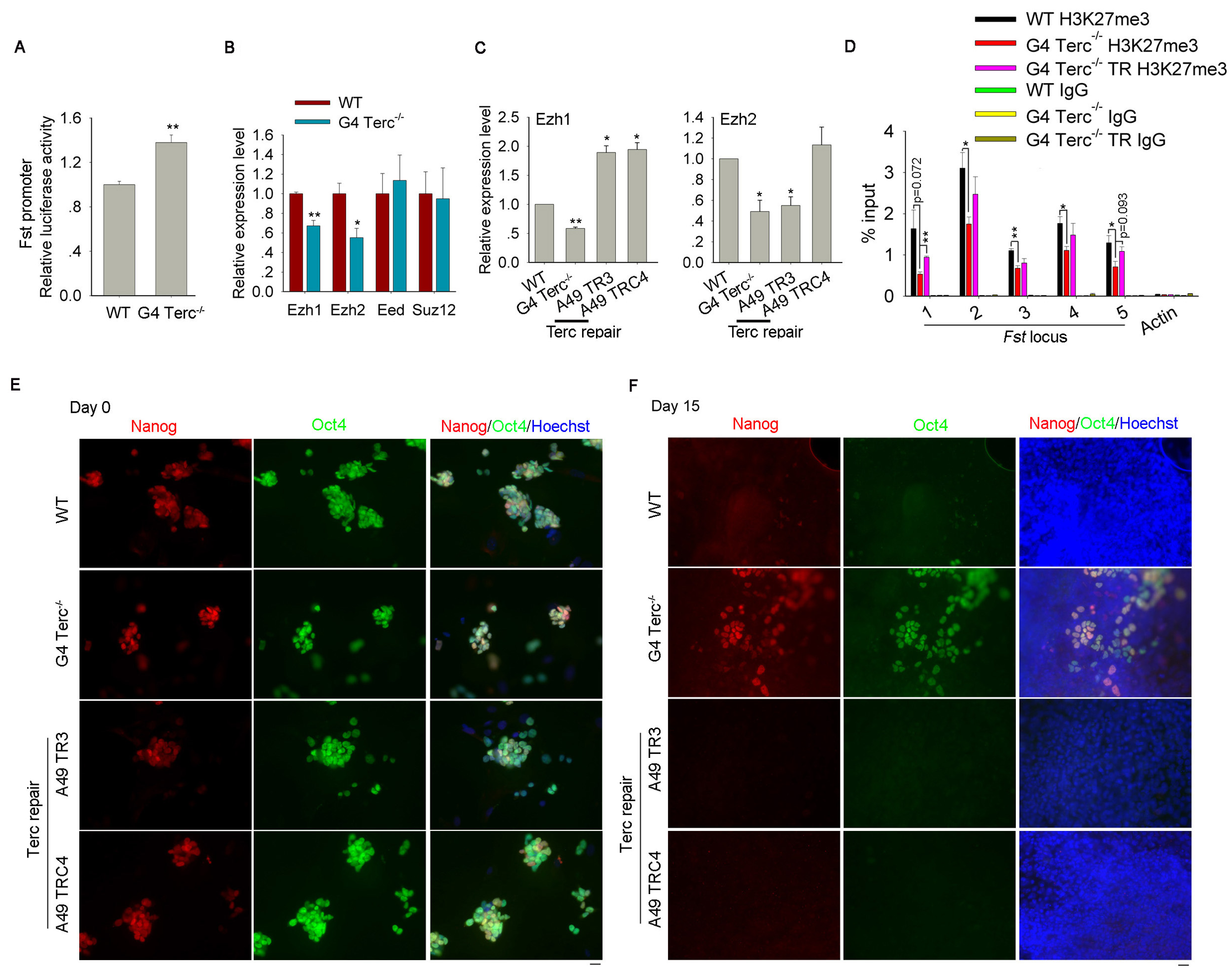

Supplement: S7 Fig — (A) Fst promoter activity is higher in G4 Terc–/–than in WT ES cells. Mean ± SEM (n = 3). **, p<0.01. (B) Relative expression levels of genes associated with PRC2 and H3K27me3 by qPCR analysis. (C) Expression levels by qPCR of Ezh1 and Ezh2 in WT, G4 Terc–/–and G4 Terc-repaired ES cells. Bars = Mean ± SEM (n = 3). *, p<0.05; **, p<0.01, compared with WT ES cells. (D) ChIP-qPCR analysis of H3K27me3 abundance at Fst promoter region in WT, G4 Terc–/–and Terc repaired ES cells, showing decreased level of H3K27me3 at Fst promoter in cells with short telomere. Mean ± SEM (n = 3). β-actin served as control. *, p<0.05; **, p<0.01. (E&F) Immunofluorescence of Nanog and Oct4 at day 0 (E) and day 15 (F) of differentiation in WT, G4 Terc–/–and G4 Terc-repaired cells. Scale bar = 20 μm. (TIF) [file pgen.1008368.s007.tif]

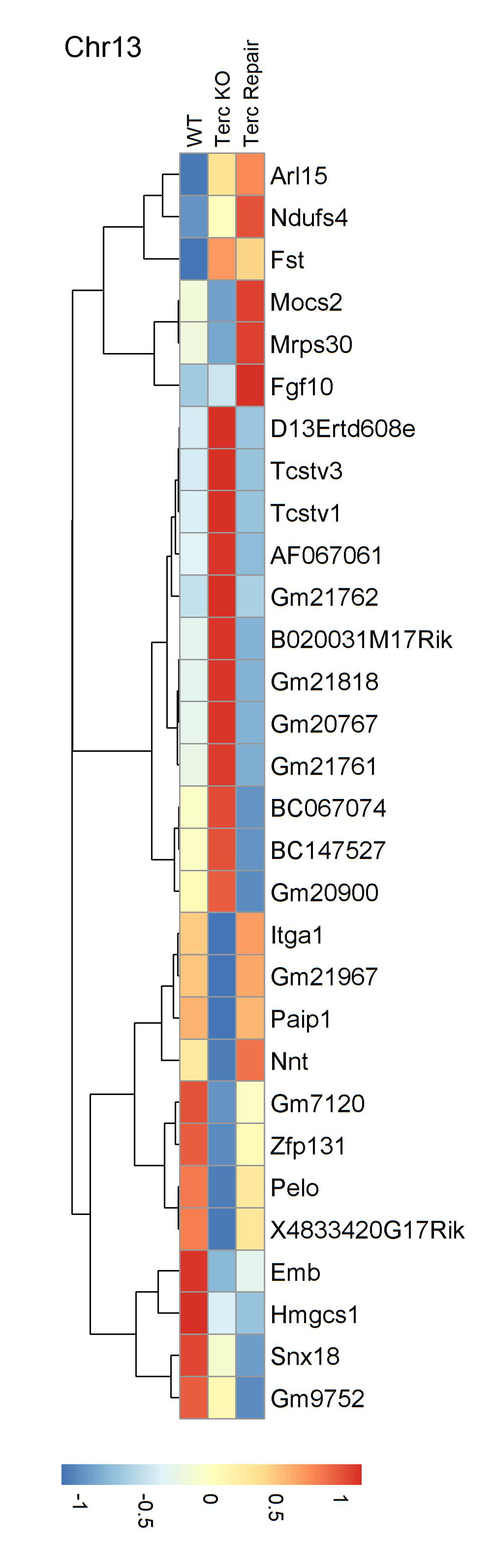

Supplement: S8 Fig — The genes near the end of long arm of chromosome 13 with expression levels with FPKM more than 1 by RNA-seq are shown. (TIF) [file pgen.1008368.s008.tif]
