# Supplementary material for: Telomere dysfunction impairs epidermal stem cell specification and differentiation by disrupting BMP/pSmad/P63 signaling
Source: PLoS Genet. 2019 Sep 13;15(9):e1008368. doi: 10.1371/journal.pgen.1008368 (PMC6760834; doi:10.1371/journal.pgen.1008368)
Supplement: S1 Table — (DOCX) [file pgen.1008368.s009.docx]

**Supplementary Table 1** Primers for quantitative real-time PCR analysis.

| **Genes** | **Forward** | **Reverse** |
| --- | --- | --- |
| Bmp4 | CTGCCGTCGCCATTCAC | CACCACCTTGTCATACTCATCC |
| Bmp7 | GCTACCACAGCAAACGCCTAA | TGCTTGGTTTCCCTTCAACA |
| Bmpr1a | AAATGGCTCGTCGTTGT | TGGAGGCTGGATTATGG |
| Bmpr1b | CAGACGGTCCTGATGCG | GCTTGCCTTGAGTGCTAA |
| Bmpr2 | CCACAACCCAGTATGCC | CGTTCCCTCCTATCTACCAA |
| Eed | ATGCTGTCAGTATTGAGAGTGGC | GAGGCTGTTCACACATTTGAAAG |
| Ezh1 | GTTCGACAGCCAGGATATGTTG | CACAAAACCGTCTCATCTTCCA |
| Ezh2 | AGTGACTTGGATTTTCCAGCAC | AATTCTGTTGTAAGGGCGACC |
| Fst | AGGGAAAGTGTATCACAAAGT | GAGTTGCAAGATCCAGAATG |
| Gapdh | TCAACAGCAACTCCCACTCTTCCA | ACCACCCTGTTGCTGTAGCCGTAT |
| Gata1 | CAGGAAGGGAAGAGCAACA | GAGAAGAAAGGACTGGGAAAGT |
| K1 | CAAGTTTGCCTCCTTCATCG | CAGCGAGTCCACCTTCCTT |
| K14 | TGCTGGATGTGAAGACAAGG | GGATGACTGAGAGCCAGAGG |
| K4 | GAGGATACGGTGCTGGGTT | CAGGCTCTGGTTGATGGTG |
| K5 | AGGCCCAGTACGAGGACATT | GCTTGTTTCTGGCATCTTTGAG |
| Nanog | TTGCTTACAAGGGTCTGCTACT | ACTGGTAGAAGAATCAGGGCT |
| Oct4 | TTGGGCTAGAGAAGGATGTGGTT | GGAAAAGGGACTGAGTAGAGTGTGG |
| p63 | GTGCCTCTACCGTCAGTG | AAGAAGACAGGAAGAACCC |
| Smad1 | ACCTGTGGCTTCCGTCTC | TCGTGGCTCCTTCGTCA |
| Smad7 | AGATGGGGGAAGTGCTTTTT | GCGTGTCCAAAAGGCTAGAG |
| Suz12 | TGCCACTAGAAATTCAGAGAGCC | TTGTGCAGGTTTAACAGAACCA |
| Terc | CATTAGCTGTGGGTTCTGGTCT | TCCTGCGCTGACGTTTGTTT |
| Tert | ACTGGTGGAGATCATCTTTCTGGG | ACCTGAGGAGTCTGACATATTGGC |
