# Supplementary material for: Telomere dysfunction impairs epidermal stem cell specification and differentiation by disrupting BMP/pSmad/P63 signaling
Source: PLoS Genet. 2019 Sep 13;15(9):e1008368. doi: 10.1371/journal.pgen.1008368 (PMC6760834; doi:10.1371/journal.pgen.1008368)
Supplement: S2 Table — (DOCX) [file pgen.1008368.s010.docx]

**Supplementary Table 2** Primers used for telomere length measurement by qPCR.

| **Gene** | **Primer** |
| --- | --- |
| mTel-Forward | CGGTTTGTTTGGGTTTGGGTTTGGGTTTGGGTTTGGGTT |
| mTel-Reverse | GGCTTGCCTTACCCTTACCCTTACCCTTACCCTTACCCT |
| 36B4-Forward | ACTGGTCTAGGACCCGAGAAG |
| 36B4-Reverse | TCAATGGTGCCTCTGGAGATT |
