# Supplementary material for: Telomere dysfunction impairs epidermal stem cell specification and differentiation by disrupting BMP/pSmad/P63 signaling
Source: PLoS Genet. 2019 Sep 13;15(9):e1008368. doi: 10.1371/journal.pgen.1008368 (PMC6760834; doi:10.1371/journal.pgen.1008368)
Supplement: S3 Table — (DOCX) [file pgen.1008368.s011.docx]

**Supplementary Table 3** Primers for methylation analysis.

| **Genes** | **Forward** | **Reverse** |
| --- | --- | --- |
| Fst | GGTAGAGTAGTAGTAGTAGGAGGTAGAGTT | ACAACCCCACACACTAAAAAAC |
| Nanog | GAGGATGTTTTTTAAGTTTTTTTT | CCCACACTCATATCAATATAATAAC |
|  | AATGTTTATGGTGGATTTTGTAGGT |  |
