# Supplementary material for: Telomere dysfunction impairs epidermal stem cell specification and differentiation by disrupting BMP/pSmad/P63 signaling
Source: PLoS Genet. 2019 Sep 13;15(9):e1008368. doi: 10.1371/journal.pgen.1008368 (PMC6760834; doi:10.1371/journal.pgen.1008368)
Supplement: S4 Table — (DOCX) [file pgen.1008368.s012.docx]

**Supplementary Table 4** shRNA sequences targeting to *Fst.*

| **Plasmid** | **Target sequence** |
| --- | --- |
| pSIREN-Control | GCGTTCAATTAGCAGACCA |
| pSIREN-Fst_T1 | AGAACGGCCGCTGCCAGGT |
| pSIREN-Fst_T3 | TGCCACATACGCCAGCGAG |
