# Supplementary material for: Telomere dysfunction impairs epidermal stem cell specification and differentiation by disrupting BMP/pSmad/P63 signaling
Source: PLoS Genet. 2019 Sep 13;15(9):e1008368. doi: 10.1371/journal.pgen.1008368 (PMC6760834; doi:10.1371/journal.pgen.1008368)
Supplement: S5 Table — (DOCX) [file pgen.1008368.s013.docx]

**Supplementary Table 5** Primers for ChIP-qPCR.

| **Promoter loci** | **Forward** | **Reverse** |
| --- | --- | --- |
| Fst-pb1 | CCCGCTCCTACGCAAATAA | \| GGTGACATTTCCCACCTTCT \| \| --- \| |
| \| Fst-pb2 \| \| --- \| | \| AGAAGACCCGCCAACTTTC \| \| --- \| | \| TTTCTGTCCCGACTCCTCT \| \| --- \| |
| Fst-pb3 | CCTCAGTAGTACATCACAGCATAA | CTCTTCCCTCCAACGCATTA |
| \| Fst-pb4 \| \| --- \| | TCGTCTTTGCAGTCGTCTTT | TCCTTATGCAGGAAGGCAAATA |
| Fst-pb5 | CAAACAGCAAAGCTCCGTATTC | CCAGAAGACCCAACACAAGAT |
| \| β-actin \| \| --- \| | CGTGTGACAAAGCTAATGAGGCTG | CTAAGTTCAGTGTGCTGGGAGTCT |
